# Mycobacterial RNase E cleaves with a distinct sequence preference and controls the degradation rates of most *Mycolicibacterium smegmatis* mRNAs

**DOI:** 10.1016/j.jbc.2023.105312

**Published:** 2023-10-05

**Authors:** Ying Zhou, Huaming Sun, Abigail R. Rapiejko, Diego A. Vargas-Blanco, Maria Carla Martini, Michael R. Chase, Samantha R. Joubran, Alexa B. Davis, Joseph P. Dainis, Jessica M. Kelly, Thomas R. Ioerger, Louis A. Roberts, Sarah M. Fortune, Scarlet S. Shell

**Affiliations:** 1 Department of Biology and Biotechnology, Worcester Polytechnic Institute, Worcester, Massachusetts, USA; 2 Program in Bioinformatics and Computational Biology, Worcester Polytechnic Institute, Worcester, Massachusetts, USA; 3 Department of Immunology and Infectious Diseases, Harvard T. H. Chan School of Public Health, Boston, Massachusetts, USA; 4 Department of Computer Science & Engineering, Texas A&M University, College Station, Texas, USA

**Keywords:** *Mycobacterium tuberculosis*, *Mycobacterium smegmatis*, *Mycolicibacterium smegmatis*, RNase E, RNA degradation, RNA processing

## Abstract

The mechanisms and regulation of RNA degradation in mycobacteria have been subject to increased interest following the identification of interplay between RNA metabolism and drug resistance. Mycobacteria encode multiple ribonucleases predicted to participate in mRNA degradation and/or processing of stable RNAs. RNase E is hypothesized to play a major role in mRNA degradation because of its essentiality in mycobacteria and its role in mRNA degradation in gram-negative bacteria. Here, we defined the impact of RNase E on mRNA degradation rates transcriptome-wide in the nonpathogenic model *Mycolicibacterium smegmatis*. RNase E played a rate-limiting role in degradation of the transcripts encoded by at least 89% of protein-coding genes, with leadered transcripts often being more affected by RNase E repression than leaderless transcripts. There was an apparent global slowing of transcription in response to knockdown of RNase E, suggesting that *M. smegmatis* regulates transcription in responses to changes in mRNA degradation. This compensation was incomplete, as the abundance of most transcripts increased upon RNase E knockdown. We assessed the sequence preferences for cleavage by RNase E transcriptome-wide in *M. smegmatis* and *Mycobacterium tuberculosis* and found a consistent bias for cleavage in C-rich regions. Purified RNase E had a clear preference for cleavage immediately upstream of cytidines, distinct from the sequence preferences of RNase E in gram-negative bacteria. We furthermore report a high-resolution map of mRNA cleavage sites in *M*. *tuberculosis*, which occur primarily within the RNase E-preferred sequence context, confirming that RNase E has a broad impact on the *M. tuberculosis* transcriptome.

Mycobacteria are a globally important group of bacteria including the pathogen *Mycobacterium tuberculosis*, which kills over a million people each year (1), as well as numerous environmental bacteria and opportunistic pathogens. Mycobacteria are phylogenetically distant from better-studied models such as *Escherichia coli*, and consequently, numerous aspects of their fundamental biology remain poorly understood. mRNA metabolism is a critical aspect of mycobacterial biology, as regulation of gene expression facilitates adaptation to stressors both during infection and in the environment, and regulation of mRNA degradation permits energy conservation during severe stress. The roles and regulation of mycobacterial mRNA degradation enzymes remain largely undefined; however, recent reports of interplay between RNA metabolism and drug resistance have highlighted the relevance of these pathways (2, 3, 4, 5, 6).

The endoribonuclease RNase E is a critical component of the bulk mRNA degradation machinery in gram-negative bacteria. In *E. coli*, RNase E cleaves single-stranded mRNAs in A/U-rich regions and interacts with other RNA degradation proteins to increase the efficiency of mRNA degradation ((7, 8, 9, 10, 11) and reviewed in Ref. (12)). In contrast, many gram-positive bacteria such as *Bacillus subtilis* and *Staphylococcus aureus* lack RNase E completely and rely on other RNases such as RNase J and RNase Y. Mycobacteria are phylogenetically more closely related to gram-positive bacteria than gram-negative bacteria, despite having cell envelopes that prevent gram staining. However, they encode orthologs of RNase E, and these genes are essential in both *M. tuberculosis* and the nonpathogenic model *Mycolicibacterium smegmatis* (13, 14, 15). The essentiality of RNase E suggests it may be a critical component of the bulk mRNA degradation machinery in mycobacteria. Consistent with this, mycobacterial RNase E was shown to interact with other RNases such as RNase J and PNPase (16). It was also shown to contribute to rRNA maturation (15).

We previously showed that the *M. smegmatis* transcriptome is shaped by endonucleolytic cleavage events that produce mRNA fragments with monophosphorylated 5′ ends (17). RNase E is known to produce cleavage products with monophosphorylated 5′ ends in other organisms. Taken together with the observation that the mycobacterial cleavage sites occurred preferentially in single-stranded regions, and the paucity of other candidate RNases predicted to cleave with those properties, we hypothesized that RNase E was responsible for the majority of the cleavage sites we mapped in *M. smegmatis*. However, the mycobacterial cleavage sites occurred primarily in a sequence context distinct from that reported to be cleaved by *E. coli* RNase E. Most mycobacterial mRNA cleavages occurred immediately upstream of a cytidine, with a preference for one to two purines immediately upstream and uridine 3 nt downstream of the cleavage site (RR↓**C**NU). A previous report tested the cleavage specificity of *M. tuberculosis* RNase E on several short substrates *in vitro*; however, none of the substrates used in that study contained the motif “RRCNU” (18).

Given the clear importance of RNase E in mycobacteria and lack of information on its role, we sought to define its function in mycobacterial mRNA metabolism. First, we used an inducible system to interrogate the effects of knockdown of *rne*, the gene encoding RNase E, in *M. smegmatis*. We found that RNase E has a rate-limiting role in degradation of most mRNAs, with a larger influence on leadered transcripts compared with leaderless transcripts. Its cleavage signature is ubiquitous across the transcriptomes of both *M. smegmatis* and *M. tuberculosis* and is distinct from that reported in gram-negative bacteria. We then used purified RNase E to confirm its cleavage specificity *in vitro*. Finally, we report a transcriptome-wide high-resolution map of major RNA cleavage sites in *M. tuberculosis*, which occur in sequence contexts corresponding to the RNase E signature. Together, our results implicate RNase E as the predominant source of 5′ monophosphorylated cleaved mRNAs in the transcriptomes of both *M. smegmatis* and *M. tuberculosis* as well as a critical mediator of bulk mRNA degradation in these organisms.

## Results

### RNase E has a global role in *M. smegmatis* mRNA degradation

**Figure 1:**
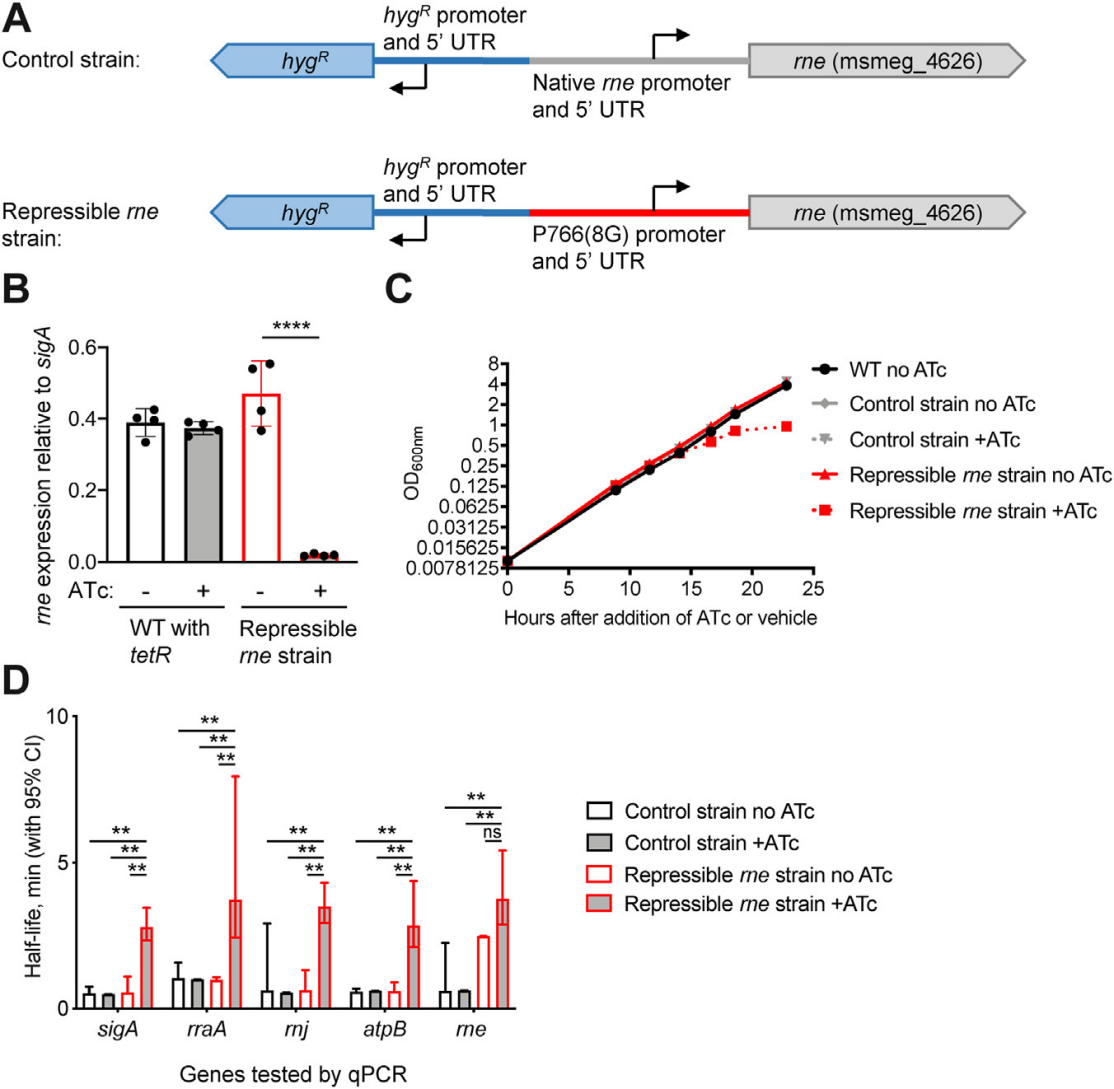
**Knockdown of *rne* expression causes growth cessation and altered transcript abundance in *Mycolicibacterium smegmatis*.***A*, promoter replacement strategy to construct a strain in which *rne* expression is repressed by addition of ATc. *B*, *rne* transcript levels were reduced in the repressible *rne* strain following 3 h of exposure to ATc. ∗∗∗∗*p* < 0.001, two-tailed *t* test. *C*, growth of the repressible *rne* strain slowed approximately 15 h after addition of ATc. *D*, 8 h after addition of ATc or vehicle, rifampicin was added to block new transcription, and mRNA levels of the indicated genes were measured at several time points by qPCR to determine their half-lives. ∗∗*p* < 0.01, pair-wise comparisons by linear regression. ATc, anhydrotetracycline; qPCR, quantitative PCR.

Given its essentiality in mycobacteria and its broad role in mRNA degradation, we sought to determine the role of RNase E in mRNA degradation transcriptome wide in a mycobacterial model. We therefore constructed an *M. smegmatis* strain in which we could repress transcription of *rne* (msmeg_4626), the gene encoding RNase E. Replacement of the native *rne* promoter and 5′ UTR (17) with the P766(8G) promoter and associated 5′ UTR (19) produced a strain in which anhydrotetracycline (ATc) caused a constitutively expressed reverse Tet repressor to bind the promoter and repress *rne* transcription (Fig. 1, *A* and *B* and Table 1). We hereafter refer to this as the repressible *rne* strain. Consistent with the known essentiality of *rne*, growth slowed approximately 15 h after addition of ATc and later ceased (Fig. 1*C*). As RNase E is untagged in our strains, we were unable to quantify depletion at the protein level. Notably, the amount of essential protein depletion required to affect growth in *M. tuberculosis* was shown to vary dramatically among essential proteins (20). Construction of the repressible strain involved insertion of a hygromycin resistance gene upstream of *rne*. We therefore constructed an isogenic strain in which the hygromycin resistance gene was inserted upstream of the native copy of *rne*, hereafter referred to as the control strain (Fig. 1*A*).

**Table 1:** Strains and plasmids used in this study

Species	Strain	Plasmid	Description	Source
*M. smegmatis*	mc^2^155	None	Widely used laboratory strain	American Type Culture Collection
*M. smegmatis*	SS-M_0424	pSS291: tetR38 driven by promoter ptb38, L5 integrating, kan^R^	mc^2^155 with the *hyg*^*R*^ gene inserted with its own promoter 347 nt upstream of, and divergent from, the *rne* translation start site	This study
*M. smegmatis*	SS-M_0418	pSS291: tetR38 driven by promoter ptb38, L5 integrating, kan^R^	mc^2^155 in which the *rne* (msmeg_4626) promoter and UTR (nt −346 through −1 relative to the *rne* start codon) were replaced by the P766(8G) promoter and associated 5′ UTR (19). In addition, the *hyg*^*R*^ gene was inserted with its own promoter upstream of, and divergent from, the P766(8G) promoter	This study
*M. tuberculosis*	H37Rv	None	Widely used laboratory strain	American Type Culture Collection
*M. tuberculosis*	H37Rv Δ*rnj*	None	The *rnj* CDS was replaced with the *hyg*^*R*^ CDS	(3)
*E. coli*	BL21 DE3 pLysS	pSS420: pET38 expressing residues 146–824 of *M. smegmatis* RNase E with N-terminal 6× His, 3× FLAG, tobacco etch virus protease cleavage site, and 4× Gly linker		This work
*E. coli*	BL21 DE3 pLysS	pSS421: pSS420 with mutations D694R and D738R		This work

While the essentiality of *rne* could be due to its role in mRNA degradation, rRNA maturation, or both, we were specifically interested in determining the role of RNase E in mRNA metabolism. We therefore evaluated the impact of *rne* knockdown on mRNA degradation rates prior to the slowing of bacterial growth. We measured the half-lives of several mRNAs by adding rifampicin (RIF) to block transcription initiation and quantifying transcript abundance at time points thereafter by quantitative PCR (qPCR). The half-life of the repressible *rne* transcript itself was longer than that of the native *rne* transcript even in the absence of ATc, but this appeared to be a feature of the transcript rather than a generalized phenomenon, as the half-lives of the transcripts of other tested genes were unaffected (Fig. 1*D*). In contrast, the half-lives of all tested transcripts were lengthened upon *rne* knockdown (Fig. 1*D*). To determine the generalizability of this observation, we used RNA-Seq to measure mRNA half-lives transcriptome wide. RNA-Seq libraries were constructed from RNA extracted from triplicate cultures of each strain and condition at various time points after the addition of RIF. qPCR was used to establish relative abundance values for a set of calibrator genes, and these were used to normalize the coverage values obtained from the RNA-Seq libraries as described in detail in the *Experimental procedures* section. Libraries were made from the repressible *rne* strain following 8 h of treatment with ATc (*rne* knockdown condition), the repressible *rne* strain in the absence of ATc, and the control strain harboring the native *rne* promoter in the presence and absence of ATc. The time point for analysis of the *rne* knockdown condition was carefully chosen to maximize our power to detect relevant phenotypes but prior to the slowing of growth. We expected growth changes would themselves affect mRNA stability as has been reported by us and many others (21, 22, 23, 24, 25, 26, 27, 28).

**Figure 2:**
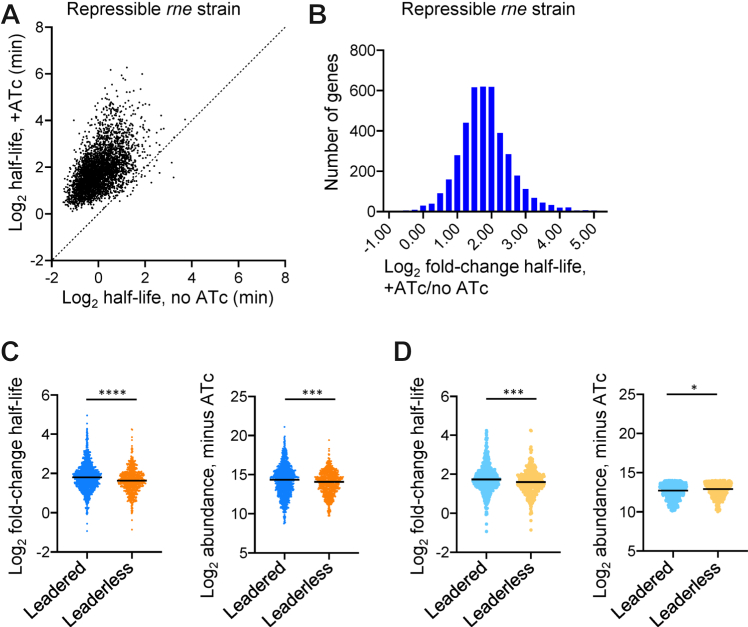
**Knockdown of *rne* expression causes stabilization of most of the *Mycolicibacterium smegmatis* transcriptome, with leadered transcripts tending to be stabilized more than leaderless transcripts.** Eight hours after addition of ATc (or vehicle) to knock down (or not) *rne*, rifampicin was added to block new transcription, and mRNA levels were measured transcriptome wide at several time points by RNA-Seq to determine half-lives. *A*, dots represent transcripts with measurable half-lives in both conditions. *B*, the distribution of fold change in half-life for the transcripts shown in *A*. *C*, the median fold change in half-life upon *rne* knockdown was higher for leadered transcripts than for leaderless transcripts (*left*). The median abundance of leadered transcripts was higher prior to *rne* knockdown (*right*). *D*, only transcripts with 10<log_2_ abundance<14 were considered, which reversed the difference in abundance trend between leadered and leaderless transcripts. The median fold change in half-life upon *rne* knockdown was still higher for leadered transcripts than for leaderless transcripts. ATc, anhydrotetracycline.

To identify transcripts that were direct targets of RNase E, we calculated half-lives for transcripts of each gene in each condition as described in the Experimental procedures section and Figs. S1–S3 (Table S1). It is important to note that the RNA-Seq libraries presumably contained mixtures of full-length mRNA and degradation products, as the RNA extraction and library construction protocols were expected to quantitatively capture most RNAs ∼≥150 nt in length. Half-lives were calculated from the summed coverage of reads across each coding sequence (CDS) at various time points following addition of RIF, and because of the relatively short reads produced by Illumina sequencing, it was not possible to distinguish reads arising from full-length transcripts *versus* degradation products. This caveat is inherent to most published transcriptome-wide studies of mRNA half-life in bacteria. We determined high-confidence half-lives for transcripts of 1643 genes and medium-confidence half-lives for transcripts of an additional 3565 genes in the *rne* knockdown condition. We were able to calculate high-confidence half-lives for 4068 of these transcripts in the repressible *rne* strain in the absence of ATc as well. Half-lives were similar in comparisons between control conditions, indicating that mRNA degradation rates were not substantially affected by the presence of ATc or by replacement of the native *rne* promoter and 5′ UTR with the Tet-repressible promoter (Fig. S4). In contrast, the half-lives of most transcripts were longer in the *rne* knockdown (Fig. 2, *A* and *B* and Table S2). The half-lives of the transcripts of 3622 genes increased by twofold or more, and the transcripts of an additional 78 genes had no measurable degradation in the *rne* knockdown. Together, these data are consistent with RNase E playing a rate-limiting step in the degradation of at least 89% of the transcriptome.

While the transcripts of most genes had longer half-lives in the *rne* knockdown condition, the magnitude of the increase in half-life varied substantially among genes (Fig. 2*B*). To investigate the factors that influence transcript sensitivity to RNase E, we examined fold-change half-life in the *rne* knockdown as a function of other potentially relevant characteristics. There was a very weak correlation between mRNA abundance in the control condition and fold change in half-life upon *rne* knockdown (Fig. S5). Previous work has reported conflicting observations about the relationship between mRNA abundance and degradation rates in bacteria. Some studies, including one on *M. tuberculosis* and several on *E. coli*, reported inverse relationships between steady-state mRNA abundance and half-lives, such that more abundant transcripts tended to be degraded more quickly (22, 23, 25, 27, 28, 29, 30). Other studies of *E. coli* and *B*. *subtilis* reported that mRNA abundance and half-life were uncorrelated or weakly positively correlated (24, 31, 32). We found a weak but statistically significant negative correlation between mRNA abundance and half-life when *rne* was expressed at normal levels, and this correlation disappeared upon *rne* knockdown (Fig. S6).

In both *M. tuberculosis* and *M. smegmatis*, approximately 15% of genes are transcribed in a leaderless fashion, meaning that transcription and translation start at the same position and there is no 5′ UTR (17, 33, 34). Other genes are transcribed as leadered genes with 5′ UTRs or in polycistronic transcripts. Leader status affects translation efficiency in different conditions and in some cases alters mRNA stability (35, 36, 37). On average, leaderless genes were less affected by *rne* knockdown than leadered genes (Fig. 2*C*, *left*). Leaderless genes also had lower median abundance than leadered genes in the control condition (Fig. 2*C*, *right*). We then considered only genes where 10 < log2 abundance <14 (Fig. 2*D*, *right*). Within this group, the median abundance of leaderless transcripts was slightly higher than that of leadered transcripts. Nonetheless, the leadered transcripts within this group still had a greater median increase in half-life upon *rne* knockdown than leaderless transcripts (Fig. 2*D*, *left*). This suggests that the difference in response of leaderless *versus* leadered transcripts to *rne* knockdown cannot be explained by differences in steady-state abundance of those transcripts. Leadered transcripts may therefore be generally more sensitive to RNase E than leaderless transcripts. However, both groups included genes that were unaffected by *rne* knockdown as well as genes that were strongly affected, indicating that additional factors are likely larger drivers of RNase E sensitivity. Given that RNase E is strongly stimulated by engagement of transcript 5′ ends in *E. coli* ((38, 39) and others), we considered that accessible 5′ ends might make transcripts more sensitive to RNase E. However, we did not find correlations between fold change in half-life upon *rne* knockdown and predicted secondary structure near the 5′ ends of transcripts (Fig. S7).

### Knockdown of *rne* affects mRNA abundance through both direct and indirect mechanisms in *M. smegmatis*

To assess the impact of *rne* knockdown on mRNA abundance, we examined transcript abundance in the *rne* knockdown strain with and without ATc prior to transcriptional blockage with RIF. These were the same samples used for the 0 min RIF treatment condition for mRNA half-life calculations, harvested 8 h after addition of ATc or vehicle control. Our normalization method allowed us to measure mRNA abundance relative to total RNA abundance, in arbitrary units. As total RNA yields were similar for all strains and conditions, this roughly approximates mRNA abundance per cell, measured in arbitrary units. A large majority of genes had increased abundance upon *rne* knockdown (Table S2). We therefore could not statistically assess differential expression using a standard pipeline such as DESeq2, for which the identification of differentially expressed genes relies on the assumption that mean gene expression is similar in the conditions being compared. Instead, we compared transcript abundance using Clipper, which does not rely on the specific data distributions of the two conditions (40). Of 6922 total genes with mean read counts >0 in both conditions, 2561 genes had increased abundance upon *rne* knockdown using cutoffs of *q* < 0.05 and fold change ≥2 (Table S3). In contrast, only nine genes that met these criteria had decreased abundance.

**Figure 3:**
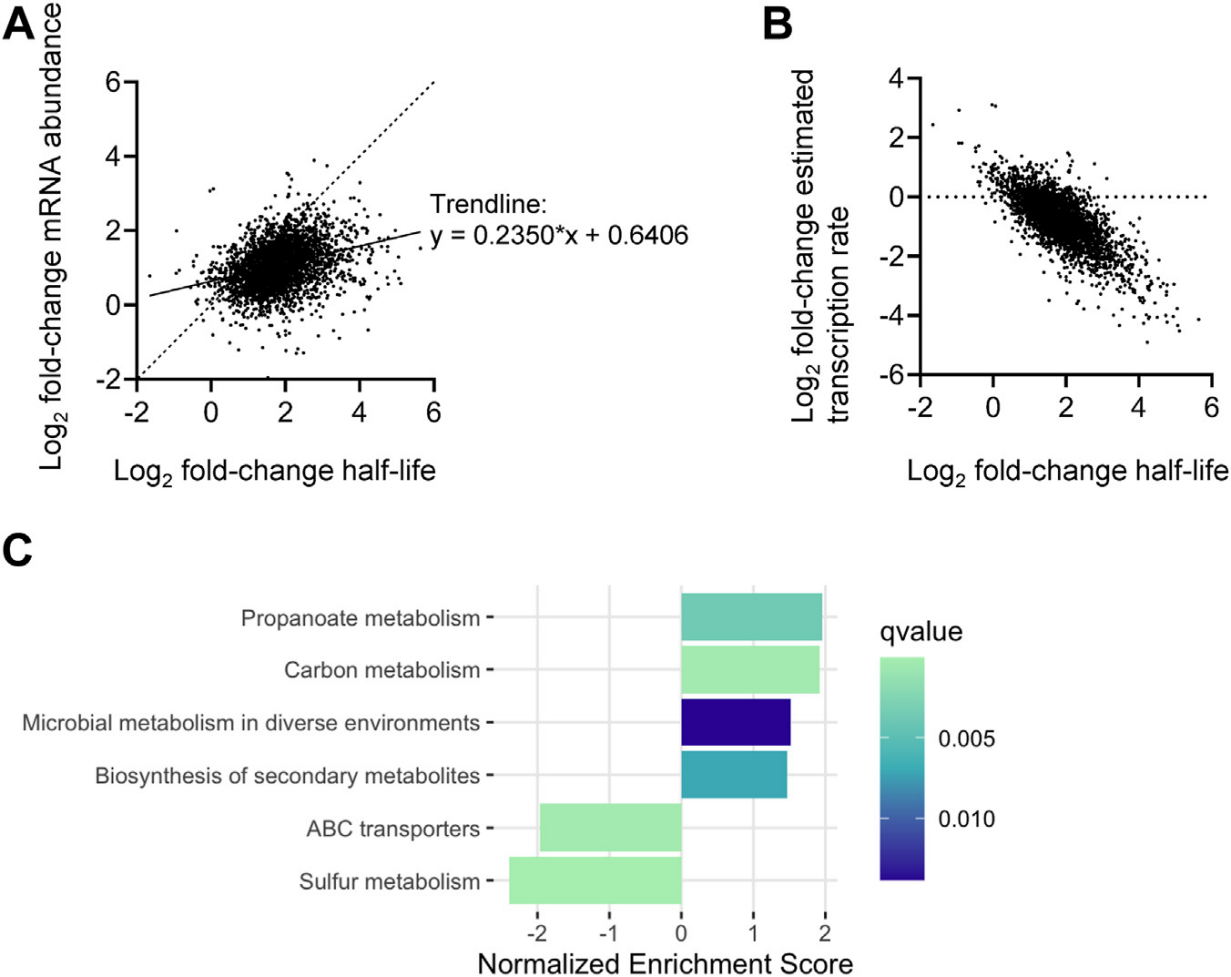
**Knockdown of *rne* impacts mRNA abundance both directly and indirectly.***A*, each *dot* represents a gene for which log_2_ fold change in transcript abundance upon *rne* repression is shown as a function of log_2_ fold change in half-life. The *solid line* shows the linear regression fit where *y* = 0.2350 ∗ *x* + 0.6406. The *dashed line* shows the expected relationship between log_2_ fold change half-life and log_2_ fold change abundance if transcription rate were unchanged. *B*, estimated transcription rates were calculated from the measured mRNA half-lives and steady-state abundance. The same genes shown in *A* are shown here. *C*, for each gene, the expected change in abundance was calculated as a function of change in half-life according to the equation in *A*. The differences between expected and observed changes in abundance were then calculated, and genes with large differences were considered more likely to be subject to active regulation. Gene set enrichment analysis was performed on the observed/expected log_2_ fold change abundance, and the gene categories with statistically significant enrichment or depletion are shown. Genes in the categories with positive enrichment scores had larger than expected increases in transcript abundance, and genes in the categories with negative enrichment scores had lower than expected increases (or had decreases) in transcript abundance. The *q* value is a *p* value corrected for multiple comparisons.

There was a significant positive correlation between increase in half-life upon *rne* knockdown and increase in abundance (Spearman's *r* = 0.3565, *p* < 0.0001; Fig. 3*A*). These observations are consistent with the idea that slower mRNA degradation leads to accumulation of mRNA in the cell. However, the changes in mRNA abundance were of a smaller magnitude than would be expected if transcription rates remained unchanged (compare the *dashed* and *solid lines* in Fig. 3*A*). We therefore used the measured mRNA abundance and half-life values to estimate transcription rates. A majority of genes had lower estimated transcription rates in the *rne* knockdown condition, suggesting the existence of a feedback process in which transcription is slowed to partially compensate for the longer mRNA half-lives (Fig. 3*B* and Table S4).

The results described previously suggested that many of the transcript abundance changes caused by *rne* knockdown were direct consequences of slower degradation that was only partially compensated for by globally reduced transcription. However, some genes did not follow the bulk trend. We hypothesized that the stress imposed by *rne* knockdown led to active transcriptional changes of some specific genes and were therefore indirect effects of *rne* knockdown. To distinguish direct and indirect transcript abundance changes, we fit the bulk relationship between log_2_ abundance change and log_2_ half-life change by linear regression to determine predicted abundance changes as a function of change in half-life (Table S2). The difference between expected and actual abundance change reflects the extent to which a gene deviated from the bulk trend. This approach makes the assumption that most abundance changes are direct. Genes with positive differences between observed and expected abundance change had higher abundance than expected upon *rne* knockdown, whereas genes with negative differences had lower abundance than expected upon *rne* knockdown. To investigate the nature of the genes that did not follow the bulk trend and therefore appeared to be actively regulated at the point of transcription in response to *rne* knockdown, we used gene set enrichment analysis (41) to identify gene categories that were overrepresented among genes with large differences between observed and expected abundances. Genes with higher-than-expected abundance were most enriched for carbon metabolism and propanoate metabolism, whereas genes with lower-than-expected abundance were enriched for sulfur metabolism and ABC transporters (Fig. 3*C*). Transcripts for the genes encoding the RNA helicase RhlE1 (msmeg_1540) and predicted RNA-binding protein KhpB (msmeg_6941) had higher-than-expected abundance, suggesting that they are transcriptionally upregulated in response to *rne* knockdown. These two proteins have reported roles as components of mycobacterial RNA degradosomes (16). It is possible that they are upregulated to partially compensate for the decrease in RNase E abundance. However, the genes encoding two other major degradosome constituents, PNPase and RNase J, did not have substantially different abundance than expected, suggesting that their abundance is not regulated in response to RNase E deficiency.

### RNase E cleavage site regions in *M. smegmatis* and *M. tuberculosis* are enriched for cytidines

**Figure 4:**
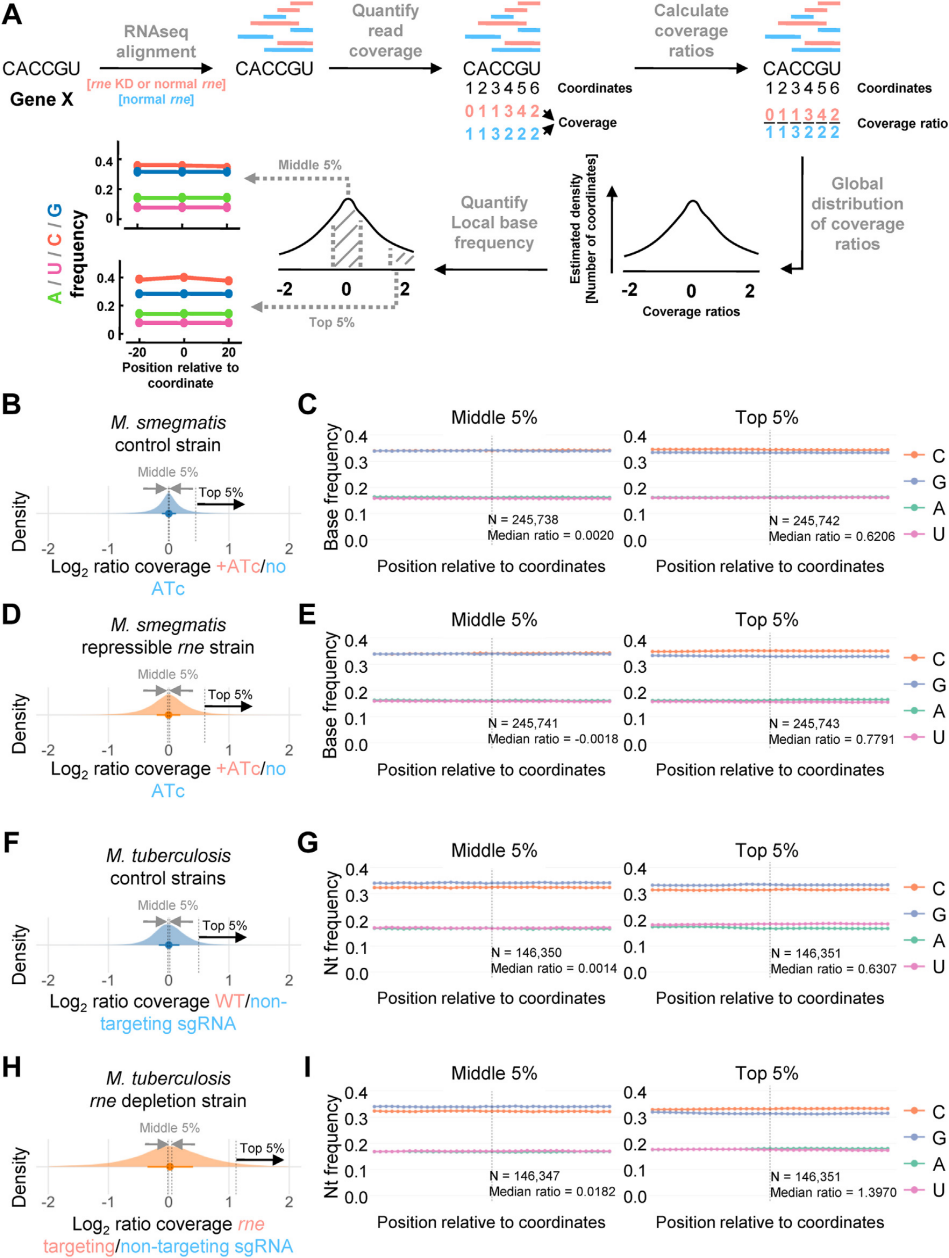
**Cytidines are enriched in regions of RNase E-dependent mRNA cleavage in both *Mycolicibacterium smegmatis* and *Mycobacterium tuberculosis*.***A*, overview of the method for relative quantification of mRNA cleavage events using standard RNA-Seq data. Illumina RNA-Seq data for both *M. smegmatis* (this work) and *M. tuberculosis* (16) were used. Both datasets included an *rne* knockdown condition and multiple control conditions. Cleavage events result in a lower proportion of reads in the immediate vicinity of the cleavage site compared with uncleaved regions of the transcript. Read depth (coverage) for each coordinate within each coding sequence across the genome was determined for each sample, then normalized by the average read depth within that gene in that sample. For each coordinate, the log_2_ ratio of coverage in the *rne* knockdown compared with a control (or two distinct controls compared with each other) was determined. The median log_2_ ratio should be approximately zero for all comparisons because of the method of normalization. Coordinates at or near RNase E cleavage sites are expected to have high ratios in the *rne* knockdown/control comparison. The regions surrounding coordinates with log_2_ ratios in the top 5% and middle 5% were then assessed for base composition bias (A, U, C, and G frequency). The bases at each position within 20 coordinates upstream and downstream of coordinates of interest (those having log_2_ ratios in the middle 5% or top 5%) were determined. *B*, Log_2_ ratios from the *M. smegmatis* control strain in the presence and absence of ATc, which is not expected to affect RNase E activity. *C*, the base frequencies in 41-nt regions centered on coordinates with log_2_ ratios in the middle 5% or top 5% of the distribution shown in *B*. *D*, Log_2_ ratios from the *M. smegmatis*-repressible *rne* strain in the +ATc condition (*rne* repressed) *versus* the no-ATc condition (*rne* expressed). *E*, the base frequencies in 41-nt regions centered on coordinates with log_2_ ratios in the middle 5% or top 5% of the distribution shown in *D*. Coordinates with log_2_ ratios in the top 5% are expected to be enriched for RNase E cleavage site–containing regions. *F*, Log_2_ ratios from two *M. tuberculosis* strains that are expected to have similar RNase E activity (a WT strain and a strain expressing a CRISPRi system with a nontargeting sgRNA). *G*, the base frequencies in 41-nt regions centered on coordinates with log_2_ ratios in the middle 5% or top 5% of the distribution shown in *F*. *H*, Log_2_ ratios from an *M. tuberculosis* strain expressing an sgRNA to knock down expression of *rne versus* a strain with a nontargeting sgRNA. *I*, the base frequencies in 41-nt regions centered on coordinates with log_2_ ratios in the middle 5% or top 5% of the distribution shown in *H*. Coordinates with log_2_ ratios in the top 5% are expected to be enriched for RNase E cleavage site–containing regions. ATc, anhydrotetracycline; sgRNA, single guide RNA.

Given the global role for RNase E implied by our data, we hypothesized that RNase E was the enzyme responsible for many of the mRNA cleavage events that we previously mapped (17). Those cleavage events occurred across the transcriptome at a sequence motif not previously associated with any RNase in any organism. The dominant feature of the cleavage site sequence context was a cytidine immediately downstream of the cleavage site. To assess the impact of *rne* knockdown on mRNA cleavage in *M. smegmatis*, we modified a recently published method for assessment of mRNA cleavage from standard paired-end RNA-Seq libraries, without construction of separate 5′-targeted libraries (42) (Figs. 4*A* and S8). This method harnesses the fact that in a standard mRNA expression library, fewer reads are obtained in regions containing cleavage sites compared with longer stretches of uncleaved RNA. When comparing the reads obtained from strains with and without knockdown of an endoribonuclease, one therefore expects to find regions of genes that have fewer reads when the RNase is expressed at higher levels. To apply this method to our *M. smegmatis rne* knockdown data, we first quantified the number of reads aligning to each coordinate within each gene in the same RNA-Seq libraries that were used for expression analyses in the previous section (0 min RIF treatment, harvested 8 h after addition of ATc or vehicle control) (Fig. 4*A*). The number of reads aligned to each coordinate is henceforth referred to as that coordinate’s coverage (Fig. 4*A*). The coverage at each coordinate in each CDS in each sample was then normalized to the summed coverage of all coordinates in that CDS to avoid confounding by genes whose mRNA abundance varied among conditions (Fig. S8). CDSs and coordinates with low coverage were filtered out. We then calculated the log_2_ ratios of coverage for each coordinate in the repressible *rne* strain in the presence *versus* the absence of ATc as well as for the control strain in the presence *versus* the absence of ATc (Fig. 4, *A*, *B* and *D*). If RNase E was responsible for cleavage at a particular site, we predicted that a smaller proportion of transcripts would exist in the cleaved form in the *rne* knockdown compared with the control conditions. We therefore expected coordinates that were very close to cleavage sites to have higher coverage in the repressible *rne* strain in the presence of ATc compared with the absence of ATc. In contrast, we did not expect coverage near RNase E cleavage sites to be affected by ATc in the control strain.

For each of the two comparisons (presence *versus* absence of ATc in the repressible *rne* strain and presence *versus* absence of ATc in the control strain), we obtained distributions of log_2_ coverage ratios for each coordinate in each gene that passed our coverage filters (Fig. 4, *B* and *D*). The distributions of log_2_ coverage ratios in the presence and absence of ATc were centered around 0 for both comparisons, because of our normalization method (Fig. 4, *B* and *D*). The distribution was broader for the *rne* knockdown strain, consistent with the expectation that RNase E levels affect the relative abundance of cleaved *versus* intact transcripts. RNase E both makes cleavage products and degrades cleavage products into pieces too small to be captured by our RNA-Seq library construction strategy; we therefore expect the effects of *rne* knockdown on the steady-state abundance of detectable cleavage products to be complex, with some cleaved RNAs decreasing in abundance and others increasing in abundance.

Nonetheless, many of the coordinates at or near RNase E cleavage sites should have high log_2_ coverage ratios for +ATc/no ATc comparison in the repressible *rne* strain, but this should not be true for the control strain where ATc does not affect RNase E levels. We used this assumption to assess the sequence context of RNase E cleavage sites by examining the sequence contexts of coordinates with high log_2_ ratios in the +ATc/no ATc comparison in the repressible *rne* strain. Specifically, we determined the sequence context of the 5% of coordinates with the highest log_2_ ratios (Fig. 4*E*). We compared this to the sequence context of coordinates with log_2_ ratios in the highest 5% in the +ATc/no ATc comparison in the control strain as well as to the sequence context of coordinates with log_2_ ratios in the middle 5% for both strains (Fig. 4, *C* and *E*). For the control strain, the relative frequencies of each base were equivalent for the coordinates with log_2_ ratios in the middle 5% and highest 5% (Fig. 4*C*), with G and C having similar frequencies that were much higher than A and U, as expected for an organism with a genomic GC content of ∼65%. In the *rne* knockdown strain, the same was true for the coordinates with log_2_ ratios in the middle 5% (Fig. 4*E*). In contrast, coordinates with log_2_ ratios in the highest 5% in the repressible *rne* strain showed a clear enrichment for cytidines (Fig. 4*E*). This is consistent with the hypothesis suggested by our previous work that RNase E has a preference for cleaving near cytidines (17). The enrichment for cytidines may appear modest compared with our previous finding that >90% of mapped cleavage events were immediately upstream of cytidines (cytidine at the +1 position). However, this modest enrichment is consistent with the nature of the method. At any given endonucleolytic cleavage site, when comparing log_2_ coverage in a strain with lower cleavage to a strain with higher cleavage, coordinates at the −1 position are expected to have equally high log_2_ ratios as coordinates at the +1 position, but only the +1 position shows a preference for cytidines. Furthermore, nearby coordinates (*e.g.*, −2, −3, −4, +2, +3, and +4) are also likely to have relatively high log_2_ ratios. Cytidines were not enriched at any position besides +1 in our previously mapped cleavage sites, and in several of those positions, there was reduced presence of cytidines (17). Adding to the complexity of the interpretation of these data, RNA-Seq expression library coverage is typically bumpy, with stochastic factors leading to variability in coverage among adjacent coordinates. The coordinates with log_2_ ratios in the highest 5% are therefore likely to include not only many −1 and +1 positions of cleavage sites but also many other coordinates that are in the general vicinities of cleavage sites. The observed modest enrichment of cytidines in Figure 4*E* is broadly consistent with the averaging of the previously observed sequence preferences in the vicinity of cleavage sites.

RNA-Seq data have been previously published for *M. tuberculosis* with *rne* knockdown (16). We therefore applied the method described previously to investigate the extent to which *M. tuberculosis* RNase E preferentially cleaves cytidine-rich regions. This was done by comparing RNA-Seq read coverage from a strain in which *rne* was knocked down by CRISPRi to coverage from a strain expressing a nontargeting CRISPRi single guide RNA (sgRNA) (Fig. 4*H*). As a control, we compared RNA-Seq read coverage from WT H37Rv to coverage from the strain expressing a nontargeting CRISPRi sgRNA (Fig. 4*F*). In the control strain comparison, the coordinates with log_2_ ratios in the middle 5% and top 5% log_2_ ratios had similar sequence contexts, which differed from the *M. smegmatis* data in having a greater proportion of guanosines than cytidines (Fig. 4*G*). This is consistent with differences in the overall nucleotide usage in the two organisms; *M. tuberculosis* CDSs contain more guanosines than cytidines, whereas *M. smegmatis* CDSs have roughly equal usage of guanosines and cytidines (Fig. S9). When comparing the strain containing an *rne*-targeting CRISPRi sgRNA to the nontargeting sgRNA strain, we found that coordinates with log_2_ ratios in the middle 5% had base frequencies similar to the control comparison, but the coordinates with log_2_ ratios in the highest 5% showed a higher frequency of cytidines compared with guanosines (Fig. 4*I*). The preference for RNase E to cleave cytidine-rich regions is therefore conserved in *M*. *tuberculosis*.

### *M. smegmatis* RNase E cleaves immediately 5′ of cytidines

To assess RNase E’s cleavage site sequence preference with higher resolution, we performed two additional analyses. First, we used 5′ rapid amplification of complementary DNA (cDNA) ends (RACE) to qualitatively compare the abundance of 5′ ends arising from a putative RNase E cleavage event in the rRNA precursor (Fig. S10*A*). We mapped a 5′ end in the spacer region between the 16S and 23S rRNAs resulting from cleavage at the sequence UG↓CU (Fig. S10*A*). Consistent with the idea that RNase E is responsible for cleaving this site, the band corresponding to the 5′ end produced by the cleavage event was fainter in the *rne* knockdown (Fig. S10, *B* and *C*). This is consistent with a previously reported role for RNase E in cleaving near this location (15), although the method used in that report did not permit precise identification of the 5′ end as we did here.

**Figure 5:**
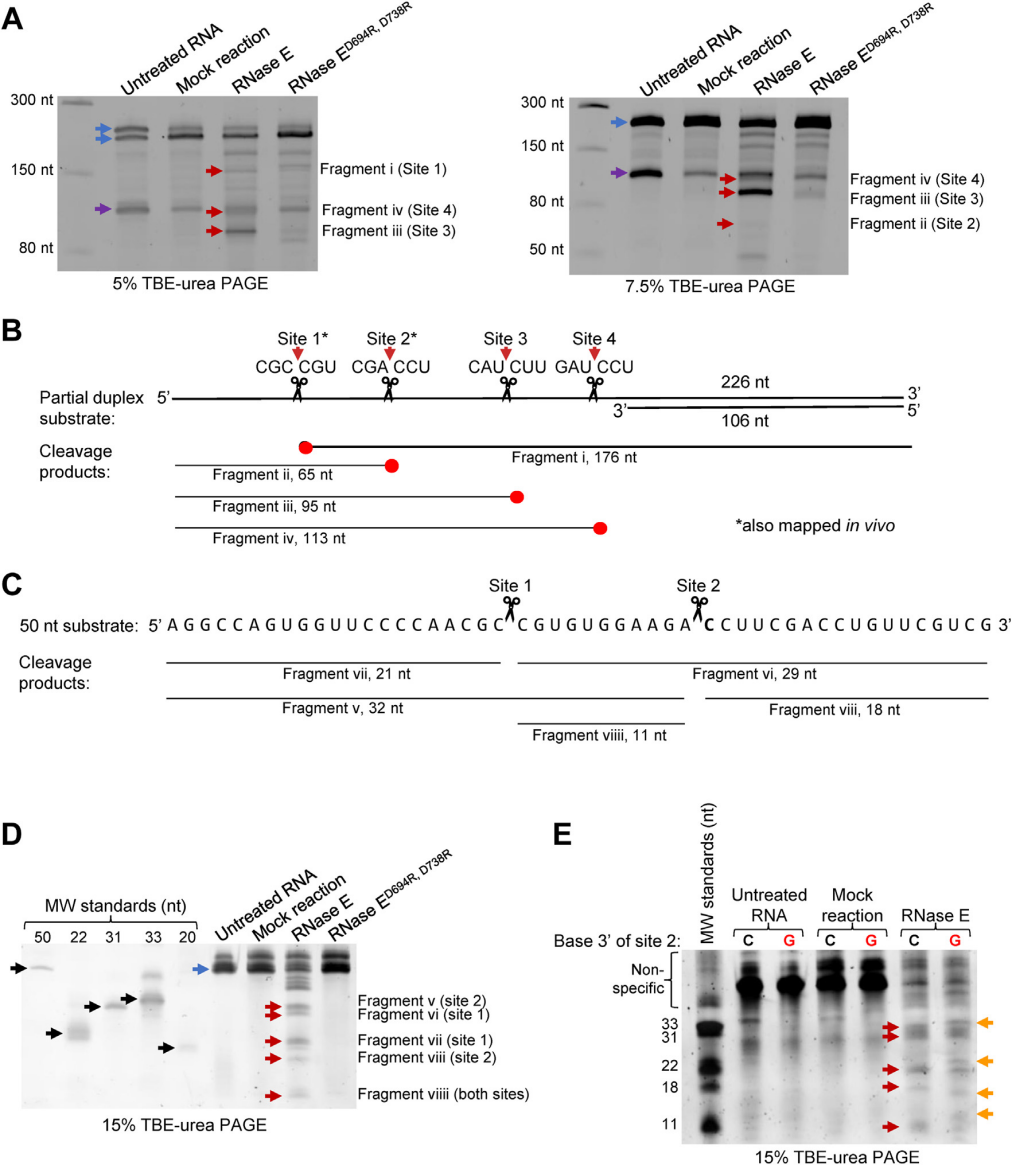
**RNase E cleaves 5′ of cytidines *in vitro*.***A*, SYBR Gold–stained Tris–borate–EDTA–urea gels revealing cleavage of the RNA substrate shown in *B* (300 ng) upon incubation for 1 h with 80 ng purified and recombinant *Mycolicibacterium smegmatis* RNase E catalytic domain (residues 146–824, with an N-terminal FLAG-His tag). The D694R, D738R mutant is predicted to be catalytically dead. Untreated RNA was not incubated with reaction buffer, whereas mock reactions contained RNA and buffer in the absence of enzyme. Cleavage sites are numbered 1 to 4, and resulting fragments visible on the gel are designated with numerals i–iv, as shown schematically in *B*. *Red arrows* denote cleavage fragments. *Blue arrows* indicate the longer strand of the partial-duplex substrate or the annealed substrate, and *purple arrows* indicate the shorter strand of the partial-duplex substrate. Note that while all samples were heated with formamide prior to loading the gels, the partial-duplex substrate did not fully denature following incubation with reaction buffer in the mock reaction or enzyme-containing reactions. *B*, schematic (not to scale) of the partial duplex RNA substrate used in *A*. *Red arrows* indicate RNase E cleavage sites mapped by 5′ or 3′ RACE on cleavage products extracted from the gels shown in *A*. The *thin lines* indicate the sizes of the extracted cleavage products (not to scale), with *red dots* indicating the ends that were mapped by RACE. *C*, a 50 nt region of the single-stranded portion of the RNA substrate shown in *A* was synthesized. The expected products from cleavage at sites 1 and/or 2 are shown. The *bolded* “C” was mutated to G in *E*. *D*, RNase E cleavage reactions using the substrate shown in *C* and enzyme that was repurified using a more stringent wash protocol to remove contaminating *E. coli* RNases. Reactions contained 80 ng RNase E and 150 ng of RNA and were incubated for 2 h. The expected cleavage products shown in *C* are indicated with *red arrows*. *Black arrows* indicate the positions of MW standards. The *blue arrow* indicates the full-length substrate. *E*, cleavage reactions were done as in *D* with the addition of a substrate with a C to G mutation at the position 3′ of cleavage site 2 (indicated with a *red* “G”). The indicated MW standards were combined in the first lane. Bands labeled “nonspecific” are unidentified byproducts of the MW standard synthesis reactions. *Red arrows* denote the expected cleavage products observed in *D*. *Orange arrows* indicate bands that appeared or shifted in position when the substrate had the C to G mutation at cleavage site 2. All gels are representative of at least three independent experiments. MW, molecular weight; RACE, rapid amplification of complementary DNA ends.

Next, we overexpressed and purified *M. smegmatis* RNase E in *E. coli* to test its cleavage specificity *in vitro*. This recombinant RNase E lacked part of the predicted N-terminal scaffold domain (deletion of residues 2–145) and most of the predicted C-terminal scaffold domain (deletion of residues 825–1037), similar to RNase E variants used for *in vitro* work in many reports (including Refs. (18, 39, 43)). Our RNase E also had N-terminal 6× His and FLAG epitope tags to facilitate purification. A variant containing the predicted catalytic site mutations D694R and D738R was purified to use as a catalytically dead control (39). The purified proteins were incubated with an *in vitro*-transcribed RNA substrate that contained a 106 bp duplex region and a 120 nt single-stranded region (Figs. 5*B* and S11). Some RNA cleavage was observed in reactions with the presumed catalytically dead RNase E, suggesting that our preps contained small amounts of an *E. coli* RNase (Fig. 5*A*). We therefore focused only on bands that appeared exclusively in reactions with catalytically active RNase E. Several of these bands were subject to 5′ and 3′ RACE to map the cleavage site locations. We mapped four distinct cleavage sites, all in the single-stranded portion of the substrate (Fig. 5, *A* and *B*). Two were at positions where we previously mapped cleavage sites *in vivo* (17), and all four occurred at the sequence motif RN↓CNU. These data confirm the propensity of RNase E to cleave single-stranded RNAs at phosphodiester bonds 5′ of cytidines.

To test the importance of having cytidines at the 3′ sides of RNase E cleavage sites, we synthesized a shorter substrate that represented 50 nt of the single-stranded region of the partial duplex used previously, containing cleavage sites 1 and 2 (Fig. 5*C*). We repurified catalytically active and dead versions of RNase E with the addition of a 1 M NaCl wash step and found the catalytically dead version had no detectable activity on the 50 nt substrate (Fig. 5*D*). In contrast, the catalytically active RNase E cleaved the substrate into bands consistent with the sizes expected from partial cleavage at both sites (Fig. 5, *C* and *D*). We then synthesized a version of the 50 nt substrate in which the cytidine on the 3′ side of cleavage site 2 was mutated to guanosine. The two bands presumed to arise from cleavage at site 2 shifted in size (Fig. 5*E*, fragments v and viii), and two new bands appeared. These results are consistent with the idea that the cleavage site cytidine is important for recognition and/or cleavage by RNase E.

### The *M. tuberculosis* transcriptome is shaped by mRNA cleavage immediately upstream of cytidines

We previously mapped *M. smegmatis* RNA cleavage sites *in vivo* by differential ligation (17). These data are complementary to the cleavage site analysis described previously; they do not give information on the RNase responsible, but they give single-nt resolution. To determine the extent to which cleavage patterns were similar in the pathogen *M. tuberculosis*, we applied the differential ligation approach. This well-validated method distinguishes between mRNA cleavage sites and primary 5′ ends produced from transcription initiation (transcriptional start sites [TSSs]) based on their different chemical properties (34, 44). We identified 2983 cleavage sites with high confidence (Table S5*A*), using a filter that required the cleaved 5′ ends to pass an abundance threshold relative to nearby expression library coverage as we did previously for *M. smegmatis*. The TSSs mapped with this approach have been reported elsewhere (34). However, the relationships between the TSSs and genes, as well as operon predictions based on TSS locations, were not previously published and are therefore reported here in Table S5, *B*–*H*.

**Figure 6:**
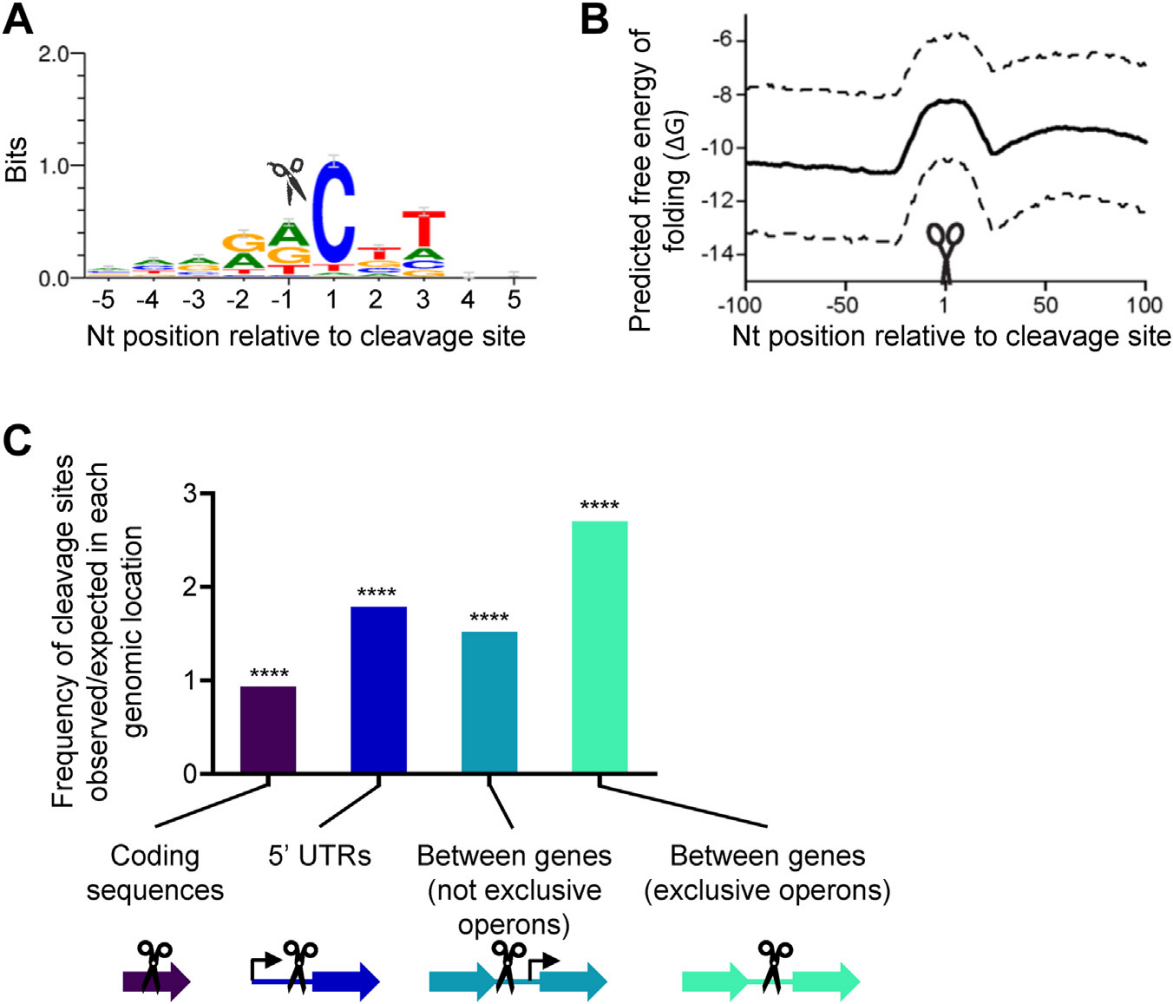
**A transcriptome-wide mRNA cleavage site map in *Mycobacterium tuberculosis* reveals sequence and secondary structure preferences consistent with RNase E and greater cleavage site frequency in 5′ UTRs and intergenic regions.***A*, WebLogo (3.7.4) generated from the complete set of mapped *M. tuberculosis* cleavage sites aligned by cleavage site position. Cleavage occurs between positions −1 and 1 as indicated by the *scissor icon*. *B*, RNA cleavage typically occurs within regions of lower secondary structure. The minimum free energy secondary structure was predicted for sliding 39 nt windows across 200 nt of sequence spanning each RNA cleavage site. For each coordinate, the mean (*solid line*; interquartile range, *dashed lines*) predicted free energy (ΔG) of secondary structure formation of all 2983 cleaved RNAs was determined. *C*, the frequencies of RNA cleavage sites in various genomic regions were determined: coding sequences, 5′ UTRs, and between adjacent genes on the same strand. Regions between genes on the same strand were separated according to whether the gene pair was predicted to be transcribed in an exclusively operonic (polycistronic) fashion. Gene pairs were considered to be transcribed exclusively in operons if only the first gene had a mapped transcription start site (TSS). Gene pairs were considered to be not transcribed exclusively in operons if each gene had its own TSS. In the latter case, genes may be transcribed as a mixture of monocistronic and polycistronic transcripts. 5′ UTRs were included only if the next upstream gene was on the opposite strand. The observed frequencies of cleavage sites in each region were compared with the frequencies that would be expected if cleavage sites were distributed among these regions without bias. ∗∗∗∗*p* < 0.0001 by binomial test comparing the observed *versus* expected frequencies.

RNA cleavage in *M. tuberculosis* occurred at a sequence motif very similar to that observed in *M. smegmatis*, with a strong bias for cleavage 5′ of cytidines (88% of high-confidence cleavage sites) and a weak bias for cleavage 3′ of purines (Fig. 6*A* and (17)). Given the multiple lines of evidence shown previously indicating that RNase E cleaves in this sequence context, we hypothesize that RNase E was responsible for most of the mapped *M. tuberculosis* cleavage sites. Analysis of the predicted secondary structure in the vicinity of cleavage sites revealed that cleavage occurred in regions more likely to be single stranded (Fig. 6*B*), consistent with expectations for RNase E (reviewed in Ref. (12)). We then removed one of the abundance filters used in the 5′ end data analysis pipeline to capture a greater number of putative cleavage sites (Table S5*I*). Analysis of the sequence context of this expanded cleavage site list revealed a similar preference for cleavage immediately upstream of cytidines (85% of the 5′ ends in the dataset) with a similar but weaker signal for sequence preferences at other positions surrounding the cleavage site (Fig. S12).

MazF was reported to also cleave near cytidines (45), but it produces 5′ hydroxyls rather than 5′ monophosphates, and its cleavage products are therefore not captured by our methodology. However, RNase J is predicted to cleave single-stranded RNAs and produce 5′ monophosphates. To determine if RNase J contributed to the mapped cleavage sites in *M. tuberculosis*, we compared the abundance of cleavage site–derived 5′ ends in a WT strain and an RNase J deletion strain (Fig. S13) (3). Most of the cleaved 5′ ends had similar abundance in the two strains, consistent with the hypothesis that most of them are not produced by RNase J.

### mRNA cleavage sites are disproportionately located in 5′ UTRs and intergenic regions in *M. tuberculosis*

To further investigate the contributors to RNA cleavage site selection in *M. tuberculosis*, we examined the frequencies of cleavage sites in CDSs, 5′ UTRs, and between adjacent genes encoded on the same strand. In each case, we assessed enrichment or depletion by comparing the observed number of cleavage sites to the number expected if cleavage was equally likely to occur in those various locations. Cleavage sites were present at less than the expected frequency in CDSs and at greater than the expected frequency in 5′ UTRs and intergenic regions (Fig. 6*C*). This pattern is similar to what we previously observed in *M. smegmatis* (17). It could be the result of differential occurrence of cleavage in these locations or could be the result of cleavage in non-CDSs being more likely to result in products stable enough to be detected. Cleavage events that trigger very rapid degradation would be unlikely to be detected by our methodology. Interestingly, the greatest enrichment for mapped cleavage sites occurred between genes that were transcribed exclusively as polycistrons (Fig. 6*C*). In some cases, there was differential abundance of the transcripts corresponding to genes upstream and downstream of the cleavage site (Table S6), suggesting that cleavage could lead to differential stability of segments of transcripts as has been reported in some other bacterial operons (46, 47, 48, 49, 50, 51, 52). Consistent with this idea, we found that two pairs of polycistronic *M. smegmatis* genes with intervening cleavage sites had differential stabilities upstream and downstream of the cleavage sites (Fig. S14).

## Discussion

Here, we used a combination of approaches to define the role of RNase E in mycobacterial mRNA degradation and identify its targets. The dramatic effect of *rne* knockdown on mRNA degradation rates in *M. smegmatis* is consistent with the essentiality of this enzyme in mycobacteria; it appears to play a rate-limiting step in degradation of the transcripts of almost 90% of genes. There was variability in the extent to which transcripts were stabilized upon *rne* knockdown, suggesting that while RNase E likely contributes to degradation of most mRNAs, other RNases may contribute differentially across the transcriptome. For example, the essential exoribonuclease PNPase could conceivably be the major degradation factor for those genes that were minimally affected by *rne* knockdown. An alternative explanation is that some mRNAs may be exquisitely susceptible to degradation, such that they were still efficiently degraded by the small amounts of RNase E present in the knockdown condition.

Most of our experiments were done 8 h after inducing repression of *rne* transcription, which was several hours prior to slowing of growth. While this strategy allowed us to distinguish the effects of RNase E knockdown from the effects of slowed growth because of loss of an essential function, we cannot distinguish with certainty which effects are direct and which are indirect. We have therefore made some assumptions in our analyses that should be noted. We assumed that the slowing of mRNA degradation was a direct consequence of reduced RNase E levels because our RNA-Seq data did not suggest that any other RNases have reduced expression. We also assumed that the impacts of *rne* knockdown on mRNA abundance were due to a combination of altered degradation (a direct effect) and altered transcription (an indirect effect). Future studies could use chemical inhibitors of RNase E (53) or degron tags (54) to fully test these assumptions and better distinguish between direct and indirect effects.

Leadered transcripts appeared to be more sensitive to RNase E levels than leaderless transcripts, suggesting that 5′ UTRs may serve as platforms for engagement with RNase E. However, there was no correlation between degree of stabilization upon *rne* knockdown and predicted secondary structure near the 5′ ends of transcripts. This suggests that the effects of 5′ UTRs on RNase E engagement cannot be explained simply by availability of 5′ ends. This finding is somewhat surprising given the reported strong effect of 5′ end engagement on RNase E activity in *E. coli* (38, 55, 56, 57) and reports of 5′ end secondary structure protecting transcripts from degradation in *E. coli* (43, 58). It is possible that mycobacterial RNase E is less 5′-end dependent than *E. coli* RNase E or that other transcript features are more important determinants of sensitivity to RNase E.

Our observation of reduced transcription upon *rne* knockdown is consistent with prior work in *E. coli* showing that transcription rate was proportional to growth rate for most genes, whereas mRNA degradation rates were inversely proportional to growth rate (22). We have found the same in *M. smegmatis*; a group of genes analyzed by qPCR had both slower degradation and lower steady-state abundance in carbon starvation and in hypoxia compared with log phase, indicating that transcription rate must be slower in the stress conditions than in log phase (21). mRNA degradation and transcription therefore appear to be coordinated in response to energy availability. While in the current work energy was not limiting and growth was not slowed, a coordination of mRNA degradation and transcription was evident. The mechanism of this coordination is unknown. Some mechanisms are known to regulate transcription in a widespread fashion in response to energy stress. For example, the stringent response represses transcription in response to starvation in *E. coli* and *B. subtilis* through distinct mechanisms (direct binding to RNA polymerase and depletion of GTP pools, respectively) and appears to have a similar function in mycobacteria (recently reviewed in Ref. (59)) although the mechanism is unknown. However, the stringent response did not affect mRNA degradation rates in *M. smegmatis* (21). Another known mechanism of global transcriptional repression in mycobacteria is upregulation of a small RNA called Ms1 in *M. smegmatis* that competes with the housekeeping sigma factor for association with RNA polymerase (60). However, Ms1 abundance was not affected by *rne* knockdown.

Our data implicate RNase E as the enzyme responsible for mRNA cleavage events that produce 5′ ends with monophosphorylated cytidines, which are widespread *in vivo* in both *M. smegmatis* (17) and *M. tuberculosis*. This cleavage sequence preference differs from what was reported in a previous study of the *in vitro* activity of *M. tuberculosis* RNase E (18). In that study, the presence of a single cytidine in an otherwise monouridine oligo was inhibitory to cleavage. However, the effects of cytidines in other sequence contexts were not tested. Our results are therefore not inconsistent with that study but rather expand upon it. The strong preference of mycobacterial RNase E to cleave 5′ of cytidines contrasts with the lack of strong base specificity by *E. coli* and *Synechocystis* sp. PCC 6803 RNase E at the +1 position ((61) and reviewed in Ref. (12)). Residue F67 in *E. coli* RNase E is highly conserved among the Proteobacteria and was proposed to play a key role in the catalytic mechanism by forming a binding pocket for the base 1 or 2 nt downstream of the cleavage site (39). Mutating this residue to Ala in *E. coli* abolished activity *in vitro* (39). However, the residue at the equivalent position in both *M. smegmatis* and *M. tuberculosis* is Val. It is tempting to speculate that differences in the key residues that position the RNA substrate in the active site are responsible for the differences in cleavage sequence preference for mycobacterial *versus E. coli* RNase E. Further work is needed to investigate this question.

Both our *in vivo* and *in vitro* data indicate that while RNase E has a strong preference for cleaving 5′ of cytidines, the impact of the surrounding sequence is weak. This could mean that the identities of the surrounding nt are unimportant for RNase E binding and cleavage or that the identities of those nt are important but act in combinatorial ways that are not obvious from the data currently available. Interpretation of the *in vivo* cleavage patterns is complicated because (1) cleavage is likely affected by ribosomes and RNA-binding proteins that protect or expose particular regions and (2) cleavage products that are rapidly degraded are not detected, and our methods therefore are biased toward identification of cleavage events that produce stable products. *In vitro*, there was a clear preference for cleavage 5′ of cytidines, and mutation of the cytidine at one cleavage site to guanosine changed the position of cleavage. However, there were many cytidines that did not produce detectable cleavage products, indicating that RNase E prefers certain positions within the test substrate. We examined secondary structure predictions of the substrate and found that the cleaved positions did not correspond to the positions most likely to be in single-stranded loops. The *in vitro* cleavage pattern therefore cannot be easily explained by the predicted secondary structure. Stem–loops near cleavage sites have been shown to stimulate or direct cleavage by *E. coli* RNase E in some contexts (62, 63, 64), and therefore, the sites cleaved in our study could be dictated in part by such *cis*-acting elements. *Cis*-acting unpaired regions have also been shown to affect cleavage by *E. coli* RNase E (65). The potential impact of the scaffold domains (which were partially deleted in our purified RNase E) should also be considered, as the *E. coli* RNase E scaffold domains were recently shown to affect catalytic activity (66).

Our study highlights the differences in the types of data obtained from different methods of RNA cleavage-site analysis as well as some of the challenges in identifying RNA cleavage sites. Ligation-based methods, as we used here for *M. tuberculosis* and as we and many others have used in the past for other bacteria, precisely reveal 5′ ends generated by RNA cleavage. However, 5′ ends are only detected from cleavage events that produce relatively stable fragments with sequence and secondary structure characteristics amenable to ligation. Fragments 5′ of cleavage sites are not captured at all; these can be captured by 3-end ligation approaches, but analysis of the resulting datasets is challenging because 3′ ends generated by many RNases (including RNases E, J, and III) are chemically indistinguishable from 3′ ends generated by transcription termination. The ligation-independent method reported previously (42) and modified here, in contrast, does not identify precise cleavage site locations but may give a broader view of the ubiquity and sequence context of cleavage sites attributable to a particular RNase engineered to be induced or repressed. Ligation-based methods may be more useful for identifying cleavage products that are stable and functional, whereas the ligation-independent approach may provide a more accurate view of the breadth of action of RNases of interest.

It is important to note that for both methods, there is no readily definable cutoff for identifying cleavage sites. It is therefore not possible to conclusively determine the total number of cleavage sites in a transcriptome using the combination of methods we have employed. Using read depth filters similar those we previously published for *M. smegmatis*, here we found ∼3000 high-confidence *M. tuberculosis* cleavage sites with the ligation-based method. Relaxing one of the filters produced a set of ∼10,000 putative cleavage sites with a similar but slightly weaker sequence context signature. Our data suggest a scenario in which the transcriptome contains many cleavage sites, some that are cleaved frequently and/or produce relative long-lived products, and others that are cleaved infrequently and/or produce relatively short-lived products. If this is true, further relaxing the filters would likely reveal still more cleavage sites, likely mixed with a greater proportion of false positives. Some sites may be cleaved so infrequently that their products are not distinguishable from noise. Together, this is consistent with (1) the underlying biology of RNases that have low sequence specificity and/or cleave at ubiquitous sequences (*e.g.*, upstream of a cytidine), (2) the fact that mRNA cleavage *in vivo* is affected by binding of macromolecules such as ribosomes and sRNAs, and (3) the reality that some cleavage products are extremely short lived and difficult to detect by any method.

It is notable that RNase J, a bifunction endonuclease/exonuclease, did not impact the abundance of most transcript 5′ ends in *M. tuberculosis*. This is consistent with the idea that RNase J has a specialized role in degradation of specific types of highly structured transcripts, as we recently reported (3), rather than a global role. It is also consistent with the idea that RNase J and RNase E may cleave similar sequences (67, 68) and therefore have partially redundant activities; however, the start contrast in phenotypes observed in mycobacterial RNase J knockout strains and RNase E knockdown strains suggests such redundancy is limited.

The cleavage sites mapped in *M. tuberculosis* were disproportionately located in untranslated regions. This may reflect the greater accessibility of such regions to RNases, as they lack protection by ribosomes. An intriguing question arising from this observation is the extent to which proteins are produced from translation of cleaved mRNAs. This has been reported in some bacteria, where there are known examples of polycistronic transcripts that are cleaved to produce fragments with different stabilities, leading in some cases to different stoichiometries of proteins encoded in operons (46, 47, 48, 49, 50, 51, 52). There is one reported example in mycobacteria, but the evidence supporting it are less conclusive (69). Further studies are therefore needed to investigate the functional consequences of stable RNA cleavage products.

## Experimental procedures

### Bacterial strains and culture conditions

*M. smegmatis* strain mc^2^155 and derivatives (Table 1) were grown in Middlebrook 7H9 liquid medium supplemented with glycerol, Tween-80, catalase, glucose, and sodium chloride as described (21) or on Middlebrook 7H10 with the same supplements except for Tween-80. *M. tuberculosis* strain H37Rv was grown in the same way with the addition of oleic acid. *E. coli* NEB-5-alpha (New England Biolabs) was used for cloning, and BL21(DE3) pLysS was used for protein overexpression. *E. coli* was grown on LB. Liquid cultures were grown at 37 °C with a shaker speed of 200 RPM, except for *M. tuberculosis*, which was shaken at 125 RPM. When indicated, ATc was used at 200 ng/ml. Antibiotic concentrations used for mycobacteria were 25 μg/ml kanamycin and 150 μg/ml hygromycin. Antibiotic concentrations used for *E. coli* were 50 μg/ml kanamycin, 150 μg/ml hygromycin, and 34 μg/ml chloramphenicol.

### *M. smegmatis* strain construction

#### SS-M_0418

The repressible *rne* strain was built by mycobacterial recombineering as described (70). A gene replacement cassette was assembled in plasmid pSS187 by NEBuilder HiFi assembly (NEB) and amplified from the plasmid as a linear fragment by PCR. The *rne* TSS is located 236 nt upstream of the translation start site (17), and the core promoter sequence is evident shortly upstream of the TSS as expected. The gene replacement cassette contained nt −846 through −347 relative to the *rne* (msmeg_4626) translation start site (a 500 bp region located upstream of the *rne* native promoter), a hygromycin resistance gene and promoter, the P766(8G) promoter that contains Tet operators (tetO), the P766(8G)-associated 5′ UTR, and the first 500 bp of *rne* CDS. About 2 μg of the gene replacement cassette were dialyzed in pure water before transformation into SS-M_0078 (WT *M. smegmatis* with the recombinase plasmid pNit-recET-Kan). Correct integration of this cassette replaced the 346 nt upstream of the *rne* translation start site with the hyg resistance gene and the P766(8G) promoter and 5′ UTR and was confirmed by sequencing. Counterselection with 15% sucrose was followed by PCR screening to identify an isolate (SS-M_0151) that lost the recombinase plasmid. SS-M_0151 was further transformed with plasmid pSS291 encoding a Tet repressor into the L5 phage integration site.

#### SS-M_0424

A hygromycin-resistant control strain was built using the method described for SS-M_0418, the difference being that the target DNA fragment that was transformed into SS-M_0078 only contained the hygromycin resistance cassette with sequence upstream and downstream of position −346 relative to the *rne* translation start site, resulting in insertion of the hyg resistance gene and promoter without deletion of any native sequence.

### RNA extraction, RNA-Seq library construction, and sequencing

Cultures were grown to an absorbance of 0.8 to 0.9, with or without addition of ATc 8 h prior, and divided into a series of 14 ml conical tubes. RIF was added to a final concentration of 150 μg/ml, and cultures were harvested after 0, 1, 2, 4, 8, 16, or 32 min by freezing in liquid nitrogen. Frozen cultures were stored at −80 °C and thawed on ice for RNA extraction. RNA was extracted (21). Illumina libraries were constructed and sequenced by the Broad Institute Microbial ‘Omics Core using the library construction procedure described (71).

### cDNA synthesis and qPCR

cDNA was synthesized as described (21) and qPCR was performed using the conditions described (21) and the primers listed in that work and in Table S6.

### Gene reannotations in *M. smegmatis* and *M. tuberculosis*

For *M. smegmatis*, we used the genome sequence of *M. smegmatis* mc^2^155 strain (NC_008596.1) from Mycobrowser Release 4 (72). For gene annotations, we combined all the annotations from PATRIC 3.6.10 (73), Mycobrowser Release 4 (72), and recently identified novel ORFs (17). The combined annotations were first updated with reannotations of 213 genes as previously described (17). Based on the assumption that transcripts starting with AUG or GUG will be translated in a leaderless fashion (34), we then further utilized the TSSs reported (17) to reannotate 156 genes whose annotated 5’ UTRs started with in-frame AUG or GUG codons. In these cases, the CDS was reannotated to start at the TSS. The resulting annotations were scrutinized to exclude duplications and genes with frame shift errors. The reannotated CDS boundaries are listed in Table S8 and were used for all further analyses unless stated otherwise.

For *M. tuberculosis*, the genome sequence and original gene annotations of *M. tuberculosis* H37Rv strain (NC_000962.3) were obtained from Mycobrowser Release 4 (72). Then for genes with only one defined TSS, we used the following procedure to determine if the CDS starting coordinates would be reannotated (34). For genes with TSS upstream of the previously annotated start codon, we reannotated the start of the CDS to the TSS for those genes with in-frame AUG or GUG at the 5′ end of the transcript. For genes that had a single TSS downstream of the previously annotated start codon, the start of the CDS was reannotated to the position of the TSS if the TSS was at an in-frame AUG or GUG within the first 30% of the previously annotated CDS. If the TSS was not at an in-frame AUG or GUG, we reannotated the start of the CDS only if the next in-frame start codon (AUG, GUG, or UUG) was found in the first 30% of the previously annotated CDS. The reannotated CDS boundaries are listed in Table S9 and were used for all further analyses unless stated otherwise.

### RNA-Seq data analysis for differential expression analysis

The 0 min RIF-treated samples were used to measure and compare steady-state transcript abundance. Reads were aligned to *M. smegmatis* mc^2^155 reference sequence NC_008596.1 from Mycobrowser Release 4 (72) with Bowtie, version 1.2.2 (74), read alignment processed by SAMtools, version 1.9 (75), counts determined by HTSeq, version 0.10.1 (76). The differential expression analysis was performed using Clipper with the gene counts normalized by qPCR normalization factors (40).

### Gene set enrichment analysis

The enrichment of Kyoto Encyclopedia of Genes and Genomes pathway was tested using ClusterProfiler, version 4.4.4 (41), based on the gene list sorted by log_2_ fold changes of expected and observed abundance. False discovery rate–adjusted *p* values were used for multiple testing correction.

### RNA-Seq data analysis for expression library–based cleavage site analysis in *M. smegmatis* and *M. tuberculosis*

This analysis was performed on the *M. smegmatis* 0 min RIF-treated samples as well as an *M. tuberculosis rne* knockdown strain and corresponding control strain ((16), Gene Expression Omnibus accession number: GSE126286). Quality control was performed using FastQC. Reads were first scanned from 5′ end to 3′ end and cut once the average quality per base of a 4-base wide sliding window dropped below 20. After such processing, reads with less than 25 bases were discarded using Trimmomatic, version 0.39 (77). Reads were aligned using Bowtie2, version 2.4.5 (78) with the “--very-sensitive” option. We first aligned reads to tRNA and rRNA sequences only. The remaining reads were aligned to NC_008596.1 (*M. smegmatis*) or NC_000962.3 (*M. tuberculosis*). *Via* SAMtools, version 1.16.1 (75), we filtered the resulting alignments by keeping only the primary alignments with MAPQ at least 10. The aligned filtered reads that mapped in proper pairs were split into their corresponding strands to quantify strand-specific coverage at the single-nucleotide level using BEDTools, version 2.30.0 (79). The coverage for each gene was then calculated by summing the single-coordinate coverage within the gene, and the average coordinate coverage for each gene was calculated by dividing the summed coverage by gene length. We only kept genes with average coordinate coverage at least five in all replicates and conditions. For those qualified genes, we excluded coordinates at overlapped gene regions for downstream analysis. To correct for the variability in expression level among genes and between conditions, we normalized single-coordinate coverages using the whole-gene coverages. The single-coordinate coverages were divided by the total summed coordinate coverage of each gene (excluding regions overlapping other genes) after adding one pseudocount to all coordinate positions. The final normalized coverage of each coordinate was the average of triplicates in each condition.

The coverage ratio at each qualified coordinate position between any two conditions was then calculated as the log_2_(condition1/condition2) ratio. For each group of coordinates under investigation (*e.g.*, coordinates with log_2_ ratios in the top 5%), we quantified the sequence context using the relative base frequency of the 20 coordinates upstream and downstream of each coordinate in the group.

### RNA-Seq data analysis for determination of half-lives in *M. smegmatis*

To calculate mRNA half-lives, data from all the time points following RIF treatment were processed. First, reads were aligned using BWA-MEM, version 0.7.17 (80). Next, the resulting alignments were processed for each strand by SAMtools, version 1.10 (75). The raw coverage of each coordinate was calculated through BEDTools, version 2.29.1 (79). Then we conducted a two-step normalization of the raw coverage. First, coverage was normalized by the total number of reads in each library. Then we calculated normalization factors by performing qPCR to determine the relative expression levels of eight genes (*sigA*, *rraA*, *esxB*, *atpE*, *rne*, msmeg_4665, msmeg_5691, and msmeg_6941; Table S7) at each sample and time point compared with the average of the 0 min RIF control strain (no ATc) samples. qPCR was done with cDNA made from random priming as described previously, separately from RNA-Seq library construction. Each qPCR was performed using 400 pg of cDNA. As ribosomal rRNA depletion was not performed, the CTs obtained from the qPCR reflect the expression level of the target gene relative to the total RNA pool, which is primarily rRNA. Normalization factors were calculated separately for the region amplified by qPCR in each of the eight genes and averaged. Specifically, for a given sample *T*_*n*_, we calculated the normalization factor *F*_*Tn*_ from the qPCR target gene expression measurements as indicated later:

Calculation of the expected RNA-Seq coverage (*T*_*n,i,RNAseq_expected*_) for each qPCR amplicon region (_*i*_) in each sample (*T*_*n*_), where *T*_*0*_ represents the average value for the control strain without ATc immediately after addition of RIF, and *qPCR* represents relative abundance of the amplicon determined by qPCR:

$$T_{n,i,RNAseq\_expected}=\left(\frac{T_{n,i,qPCR}}{T_{0,i,qPCR}}\right)*\;T_{0,i,RNAseq\_actual}$$

Calculation of a global normalization factor (*F*_*Tn*_) by calculating and averaging the normalization factors for each qPCR amplicon region:

$$F_{T_{n}}=\frac{1}{8}\sum_{i=1}^{8}\frac{T_{n,i,RNAseq\_expected}}{T_{n,i,RNAseq\_actual}}$$

Then the final normalized coverage for each coordinate was calculated by multiplying the first step normalized coverage by the global normalization factor for each sample. The coverage for each gene was then represented by the summation of the normalized coverage of its coordinates, divided by the gene length.

### Estimation of transcription rates

Estimated transcription rates were calculated as a function of steady-state abundance and mRNA degradation rate as described (36) and as follows:

Transcription rate = VT = (k∗mRNA) + (μ∗mRNA)

mRNA = steady-state mRNA abundance (taken from 0 min RIF treatment)

k = degradation rate = ln (2)/half life

μ = growth rate = ln (2)/doubling time.

Doubling time = 150 min.

The estimated transcription rate units are arbitrary and therefore useful only for comparison of genes or conditions within this study.

### mRNA cleavage site mapping in *M. tuberculosis*

Mapping of *M. tuberculosis* TSSs was previously described (34). The same dataset was used to identify mRNA cleavage sites. All analyses of this dataset were done using the genome annotations in NC_000962.gbk rather than the reannotations shown in Table S9. As described in (34), RNA 5′ ends were identified, filtered based on absolute read depth and read depth relative to local expression library coverage, and subject to Gaussian mixture modeling to distinguish between TSSs and cleavage sites on the basis of relative coverage in libraries from RNA treated with RppH (“converted,” capturing both TSSs and cleavage sites) and libraries from untreated RNA (“nonconverted,” capturing primarily cleavage sites). The 5′ ends with converted/nonconverted library read depth ratios less than 1.39 had a cumulative probability of ≤0.01 of belonging to the TSS population (after adjusting for multiple comparisons by the Benjamini–Hochberg procedure) and were therefore designated RNA cleavage sites. Because cleavage may be imprecise, filtering was performed to retain the single cleavage site with the greatest converted-library read coverage in each 5 nt window. This resulted in the 2983 high-confidence cleavage sites reported in Table S5*A*. The longer list of putative cleavage sites reported in Table S5*I* was obtained by applying the same converted/nonconverted ratio cutoff to a list of 5′ ends from earlier in the pipeline prior to filtering on coverage relative to local expression library coverage. Instead, only a filter requiring a minimum mean converted library read depth of 20 was applied. This resulted in 10,795 putative cleavage sites.

### *M. tuberculosis* TSS analyses

TSSs from the aforementioned dataset were considered to be associated with the 5′ ends of genes if they were either (1) within 500 nt upstream of an annotated start codon or (2) within the first 25% of an annotated CDS. TSSs were considered to be internal within CDSs if they were located between 25% and 80% of the way through annotated CDSs. TSSs were considered to be associated with putative antisense transcripts if they did not meet any of the aforementioned criteria and were either (1) located on the opposite strand of an annotated CDS or (2) located <200 nt from the end of an annotated CDS on the opposite strand. TSSs were considered to be intergenic if they did not meet any of the aforementioned criteria for 5′-end associated, internal, or antisense transcripts.

Genes were assigned to operons if they were transcribed consecutively on the same strand and if both the following criteria were met: (1) Only the first gene had an assigned TSS and (2) the downstream gene(s) were sufficiently expressed. Sufficient expression was defined as having a reads per kilobase of transcript per million mapped reads value in corresponding RNA-Seq expression libraries equal to the fifth percentile or above of reads per kilobase of transcript per million mapped read values for all genes with TSSs. This prediction algorithm is conservative and excludes many loci that may be transcribed both polycistronically and monocistronically.

### Analysis of *M. tuberculosis* cleavage site locations relative to genes

We determined the number of coordinates in the *M. tuberculosis* genome that fell into each of the following four categories of regions: (1) CDSs; (2) 5′ UTRs of genes with mapped TSSs and for which the next upstream gene was encoded on the opposite strand; (3) regions between the CDSs of two consecutive genes encoded on the same strand for which both genes had mapped TSSs (not exclusive operons); and (4) regions between the CDSs of two consecutive genes encoded on the same strand for which only the first gene had a mapped TSS (exclusive operons). We then determined the number of cleavage sites that were located within each of these regions. The expected frequency of cleavage sites in each region was defined as:

(number of coordinates in region/sum of coordinates in all four regions) ∗ total number of cleavage sites in all four regions.

The observed number of cleavage sites in each region was then divided by the expected number to obtain the values plotted in Figure 6*C*.

### Secondary structure prediction

Free energy of RNA folding and basepair probabilities for minimum free energy structure were predicted using the Vienna RNA Package utility RNAfold (81). For Figure 6*B*, the 200 nt region spanning each RNA cleavage site was extracted, and the minimum free energy of secondary structure formation was predicted for 39 nt sliding windows across each such region. The data plotted are the mean and the 25th and 75th percentile minimum free energies of 39 nt windows centered around each relative coordinate in all cleaved RNAs.

### 5′ RACE to map a putative RNase E cleavage site in the rRNA transcript

Enzymes were obtained from New England Biolabs unless otherwise specified. Five hundred nanograms of each RNA sample were mixed with 1 μg of oligo SSS1016 in a total volume of 9 μl, incubated at 65 °C for 10 min, and cooled on ice for 5 min. Each sample was combined with 21 μl of ligation mix containing 10 μl of 50% PEG8000, 3 μl of 10 T4 RNA ligase buffer, 3 μl of 10 mM ATP, 3 μl of dimethyl sulfoxide (DMSO), 1 μl of murine RNase inhibitor, and 1 μl of T4 RNA ligase. Samples were incubated at 20 °C overnight and purified with a Zymo RNA Clean & Concentrator-5 kit according to the manufacturer’s instructions with the following modifications: samples were first diluted by addition of 20 μl of RNase-free water, and samples were eluted in 8 μl of RNase-free water. Three microliters of each purified ligation were then subject to cDNA synthesis or mock (no-RT) cDNA synthesis. Samples were combined with 1 μl of a mix containing 50 mM Tris (pH 7.5) and 500 ng/μl random primers (Invitrogen), incubated at 70 °C for 10 min, and snap-cooled in an ice-water bath. cDNA synthesis was done as described (21). About 35 ng of cDNA or the equivalent volume of the corresponding no-RT sample were mixed with 2.5 μl 10× Taq buffer, 1.25 μl each 10 μM primers SSS1017 and SSS2210, 1.25 μl DMSO, 0.5 μl of 10 mM each dNTP mix, 0.167 μl Taq polymerase, and water to a final volume of 25 μl. Cycling conditions were 5 min at 95 °C, 35 cycles of 30 s at 95 °C, 20 s at 52 °C, and 25 s at 68 °C, and a final 5 min incubation at 68 °C. PCRs were run on 1.5% agarose gels, and bands that appeared in cDNA samples but not in no-RT samples were excised and sequenced with SSS2210 to identify the adapter–RNA junctions.

### Overexpression and purification of recombinant RNase E variants

Two RNase E variants were recombinantly expressed and purified for *in vitro* RNA cleavage assays: residues 146 to 824 (partial N-terminal truncation and full C-terminal truncation), and residues 146 to 824 with D694R and D738R mutations. pSS348, carrying the *M. smegmatis rne* CDS with a Δ1-145aa partial N-terminal deletion, Δ825 to 1037aa full C-terminal deletion, and an N-terminal addition of 6× His tag, 3× FLAG tag, tobacco etch virus protease cleavage site, and 4× Gly linker sequences, was used as a template for creation of pSS420, which encodes RNase E residues 146 to 824 with the indicated tags in a pET38 backbone. pSS420 was then used as a template for creation of pSS421, which has the mutations D694R and D738R, predicted to abolish catalytic activity (39). All constructs were sequenced to confirm the success of point mutations and truncations.

*E. coli* strain BL21(DE3)pLysS was transformed with each of the RNase E expression plasmids, and 500 to 1000 ml cultures were grown to an absorbance of ∼0.5 at 600 nm, then induced with 400 μM IPTG, and incubated at 28 °C for 4 h prior to harvest. For the protein used in Figure 5*A*, pellets were resuspended in 1× immobilized metal ion affinity chromatography buffer (20 mM Tris–HCl [pH 7.9], 150 mM NaCl, 5% glycerol, and 0.01% Igepal) containing 10 mM imidazole and lysed with a BioSpec Tissue-Tearor (10 cycles of 15–30 s each at maximum speed, with 30–60 s on ice between cycles). Lysates were cleared by centrifugation, incubated for 30 to 60 min on ice with 4 ml His-Pur nickel–nitrilotriacetic acid resin 50% slurry (Thermo Scientific), washed with IMAC buffer containing 10 mM imidazole, and eluted with IMAC buffer containing 150 mM imidazole. For the proteins used in Figure 5, *D* and *E*, the NaCl concentration in the lysate was increased to 1 M before mixing with resin pre-equilibrated in the same, and the wash buffer contained 1 M NaCl. The lysis buffer also included 1× Halt Protease Inhibitor Cocktail, EDTA-Free (ThermoFisher), 40 mg of lysozyme, and 16 U Turbo DNase (Invitrogen). Eluates were concentrated with Microcon PL-30 (30,000 NMWL) protein concentrators (MilliporeSigma) and loaded onto 1 cm diameter, 38 ml Sephacryl S-200 High Resolution resin (GE Healthcare) size-exclusion chromatography columns. Flow rate was regulated using a Masterflex C/L pump. The buffer was 1× IMAC with the addition of 1 mM EDTA and 1 mM DTT.

### Preparation of *in vitro*-transcribed RNA substrates

Genomic DNA was used as a template to produce PCR products containing portions of the *atpB-atpE* locus downstream of the T7 Phi2.5 promoter and sequence needed for A-initiated transcription (TAATACGACTCACTATT**A**GG, where transcription initiates at the bolded “A”). One PCR product had the promoter oriented to produce the sense strand, and the other was shorter and had the promoter oriented to produce a partial antisense strand (Fig. S11). Monophosphorylated RNA was synthesized from each of these PCR products in the presence of a 50-fold molar excess of AMP over ATP (82) with T7 RNA polymerase (NEB; catalog no.: M0251). Each 50 μl reaction contained 1× reaction buffer, 5 mM DTT, 1 mM UTP, 1 mM CTP, 1 mM GTP, 0.5 mM ATP, 25 mM AMP, 5 units/μl T7 RNA polymerase, 1 unit/μl murine RNase inhibitor, and 2 μg DNA template. Reactions were incubated at 37 °C for 16 h. The resulting transcripts were treated with TURBO DNase at 37 °C for 30 min before purification with a Zymo RNA Clean & Concentrator-5 kit.

The *atpB-E* sense transcript and antisense transcript were combined at a 1:1 M ratio, and the mixtures were incubated in the presence of 5× annealing buffer (50 mM Tris–HCl, pH 7.9, 0.5 mM EDTA, pH 8.0, 100 mM NaCl) in a 10 μl reaction for 1 min at 90 °C, then slowly cooled down to room temperature over a period of approximately 30 min. The resulting annealed RNA mix was immediately stored at −80 °C.

The 50 nt substrate (Fig. 5*C*) was synthesized using the same conditions, except the *in vitro* transcription templates were annealed oligos (Table S7) rather than PCR products. Smaller molecular weight standards (Table S7) were also made by *in vitro* transcription from annealed oligos. About 25 μM of each of the two DNA oligos were incubated in annealing buffer (10 mM Tris, 50 mM NaCl, and 1 mM EDTA) at 95 °C for 2 min, followed by 47 cycles of 1.5 min starting at 95 °C and decreasing by 1.5° per cycle.

### *In vitro* RNase E cleavage reactions

*In vitro* RNase E cleavage reactions were heated at 65 °C for 3 min prior to adding the enzyme, then cooled, and incubated at 37 °C for 1 to 2 h following addition of the enzyme. The reaction buffer was composed of 20 mM Tris–HCl, pH 7.9, 100 mM NaCl, 5% glycerol, 0.01% IGEPAL, 0.1 mM DTT, 10 mM MgCl_2_, and each reaction containing 150 to 300 ng annealed RNA mix and 80 ng of purified RNase E. For the reactions shown in Figure 5, *D* and *E*, the buffer included 10 μM ZnCl_2_. For mock reactions, water was used instead of enzyme. Reactions were stopped by adding equal volumes of 2× Invitrogen Gel loading buffer II and then subjected to electrophoresis on a 15%, 7.5%, or 5% polyacrylamide–8 M urea gels and visualized after 15 min staining with SYBR Gold Nucleic Acid gel stain. When indicated, bands of interest were excised, and RNA was recovered using Zymo small-RNA PAGE recovery kit for 5′ RACE or 3′ RACE.

### 5′ RACE and 3′ RACE to map cleavage sites from *in vitro* RNase E cleavage reactions

For 5′ RACE, RNA extracted from bands as described previously was mixed with 1 μg of RNA oligo SSS1016 in a total volume of 9 μl at 65 °C for 5 min, chilled on ice, and then combined with 30 U T4 RNA Ligase 1 (NEB; catalog no.: M0437M), 40 U murine RNase inhibitor (NEB), 10% DMSO, 1 mM ATP, 1× T4 RNase Ligase 1 reaction buffer, and 16.7% PEG 8000 in reactions with a total volume of 30 μl. Reactions were incubated at 20 °C for 18 h followed by column purification. cDNA was synthesized using the reverse oligo SSS916, which anneals close to 3′ end of the sense strand and the cDNA synthesis protocol described previously. cDNA was purified and then was used as template to perform Taq PCR with primers SSS1018 and SSS916. Purified PCR products were sequenced with oligo SSS916.

For 3′ RACE, RNA extracted from bands as described previously was mixed with 1 μg RNA oligo SSS2433 (which has a 5′ monophosphate and a 3′ inverted deoxythymidine and was modified from Ref. (83) at 65 °C for 5 min, chilled on ice, and incubated at 17 °C for 18 h with the same reaction mix as used for 5′ RACE previously. Following column purification, cDNA was synthesized using reverse oligo SSS2434, which anneals to the 3′ adapter, and the protocol described previously. cDNA was purified and then was used as template to perform Taq PCR with primers SSS917 and SSS2434. Purified PCR products were sequenced with oligo SSS917.

### Statistical analyses and scripts

Statistics shown in Figures 1, 2 and 6 were done in GraphPad Prism, version 9.2.0 (GraphPad Software, Inc).

## Data availability

All RNA-Seq data generated in this study are available at GSE227248. The scripts for RNA-Seq processing, analysis, and result visualization are available on Github (https://github.com/ssshell/Mycobacterial_RNase_E).

## Supporting information

This article contains supporting information (15, 81, 84).

## Conflict of interest

The authors declare that they have no conflicts of interest with the contents of this article.
