# Supplementary material for: Mycobacterial RNase E cleaves with a distinct sequence preference and controls the degradation rates of most Mycolicibacterium smegmatis mRNAs
Source: J Biol Chem. 2023 Oct 5;299(11):105312. doi: 10.1016/j.jbc.2023.105312 (PMC10641625; doi:10.1016/j.jbc.2023.105312)
Supplement: Supplemental Figures [file mmc10.pdf]

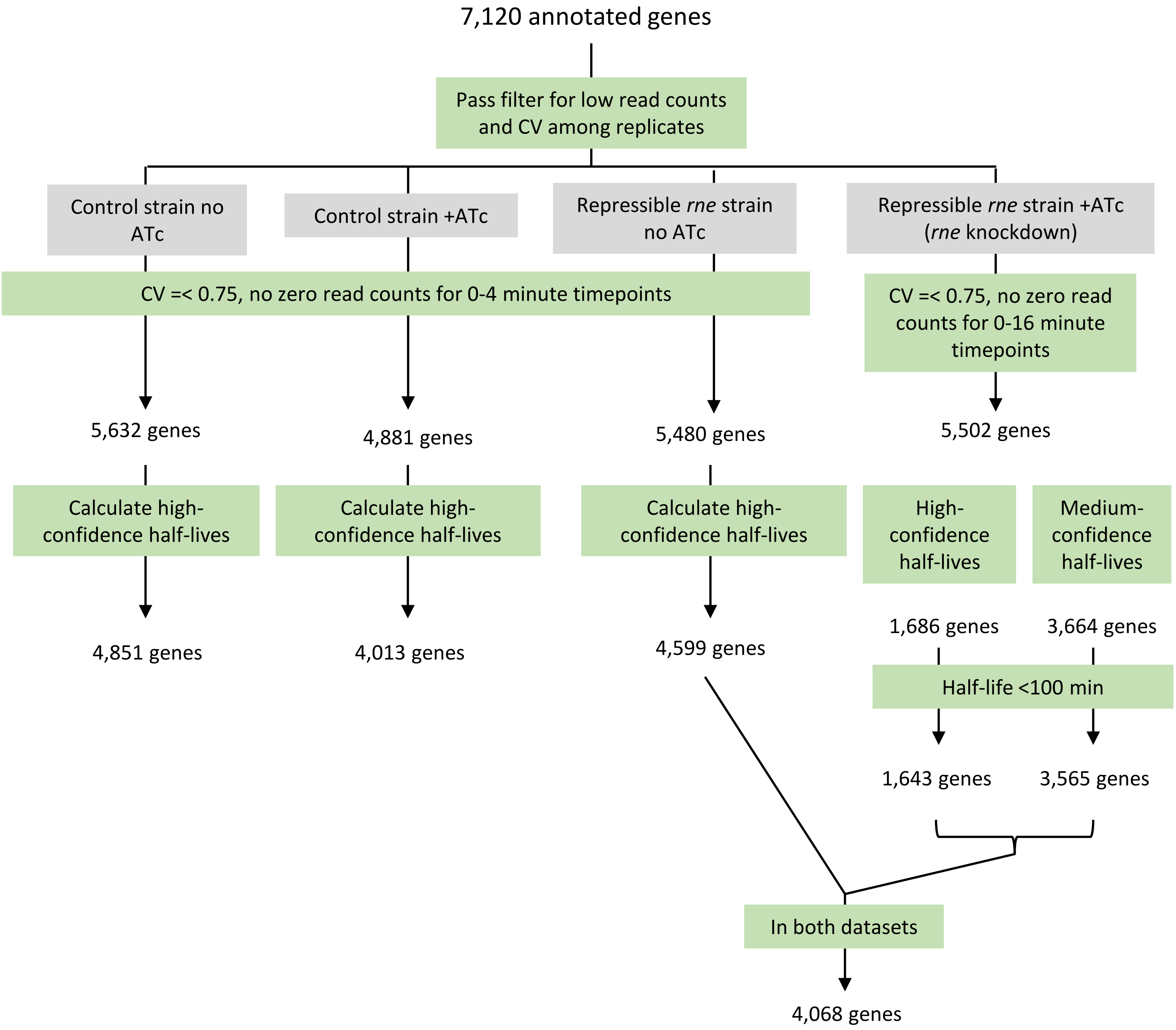

Figure S1. Overview of RNAseq data filtering for half-life calculations. Genes were used when they passed filters for read depth and CV among replicates. Half-life calculations are diagrammed in figures S2 and S3.

A

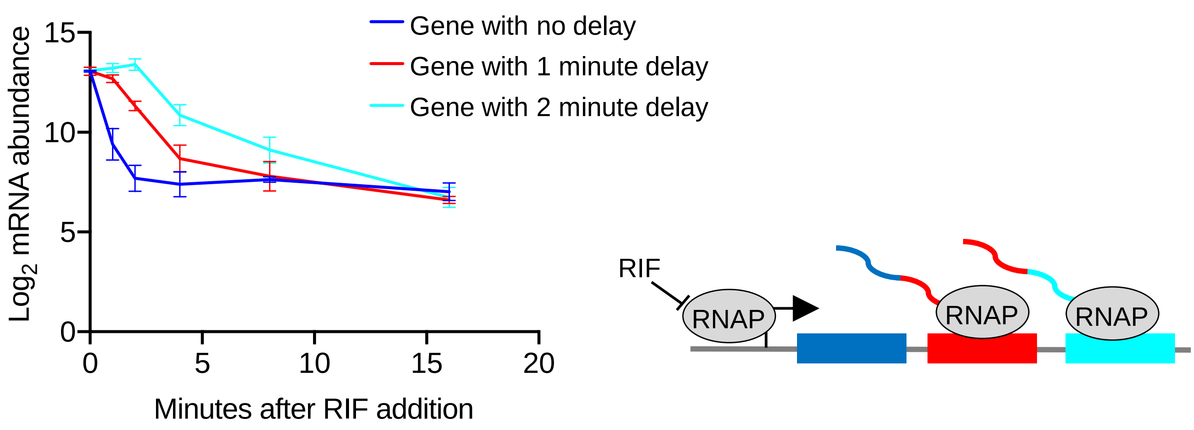

B

No delay
Genes that do not meet criteria for 1- or 2-minute delay below

→ Choose consecutive timepoints with the lowest MSE (must be < 0.5):
0-1-2 min
0-1-2-4 min
0-1-2-4-8 min

1-minute delay, category 1
Genes that meet all the following criteria:

(slope 0 to 1 min) – (slope 1 to 2 min) > 0.3
(slope 0 to 1 min) – (slope 0 to 4 min) > 0.3
(slope 1 to 2 min) – (slope 2 to 4 min) < 0.5
abundance at 2 min < abundance at 0

1-minute delay, category 2
Genes that meet all the following criteria:

(slope 0 to 1 min) > -0.1
(slope 0 to 1 min) – (slope 1 to 2 min) > 0.2
(slope 0 to 1 min) – (slope 1 to 4 min) > 0.2
Does not meet criteria for 2-minute delay

→ Choose consecutive timepoints with the lowest MSE (must be < 0.5):
1-2-4 min
1-2-4-8 min
1-2-4-8-16 min

2-minute delay, category 1
Genes that meet all the following criteria:

(slope 0 to 2 min) > -0.3
(slope 0 to 2 min) – (slope 2 to 4 min) > 0.25
Does not meet criteria for 1-minute delay, category 1

2-minute delay, category 2
Genes that meet all the following criteria:

(slope 0 to 2 min) – (slope 2 to 8 min) > 0.2
Does not meet criteria for 1-minute delay, category 1

→ Choose consecutive timepoints with the lowest MSE (must be < 0.5):
2-4-8 min
2-4-8-16 min

→ Half-life = -1/slope

Figure S2. Half-life calculation procedure for genes in control conditions (*rne* not repressed). (Continued on following page)

Figure S2. Half-life calculation procedure for genes in control conditions (*rne* not repressed). (Continued from previous page) **A.** Log_2-transformed mRNA abundance data show three distinct degradation patterns following addition of rifampicin to block transcription. Because rifampicin blocks transcription initiation but not transcription elongation, some genes show a delay before transcript levels decrease. The delay generally corresponds to the distance between the gene and its transcription start site. Furthermore, degradation for all genes reaches a plateau at later timepoints. We expect that the linear portion of the degradation curve between the delay (if present) and plateau is most likely to reflect the true degradation rate and therefore use this to calculate the half-life. **B.** Classification of genes into three delay categories based on linear regression fits to different sets of timepoints following addition of rifampicin, followed by half-life determination. The slopes between the indicated timepoints were calculated and used to classify genes as having no delay, a 1-minute delay, or a 2-minute delay. After removal of early timepoints as indicated to account for the delay, the mean squared error (MSE) was used to quantify goodness of fit for linear regression using subsets of the remaining timepoints and the set of timepoints with the best fit were used to calculate the half-life.

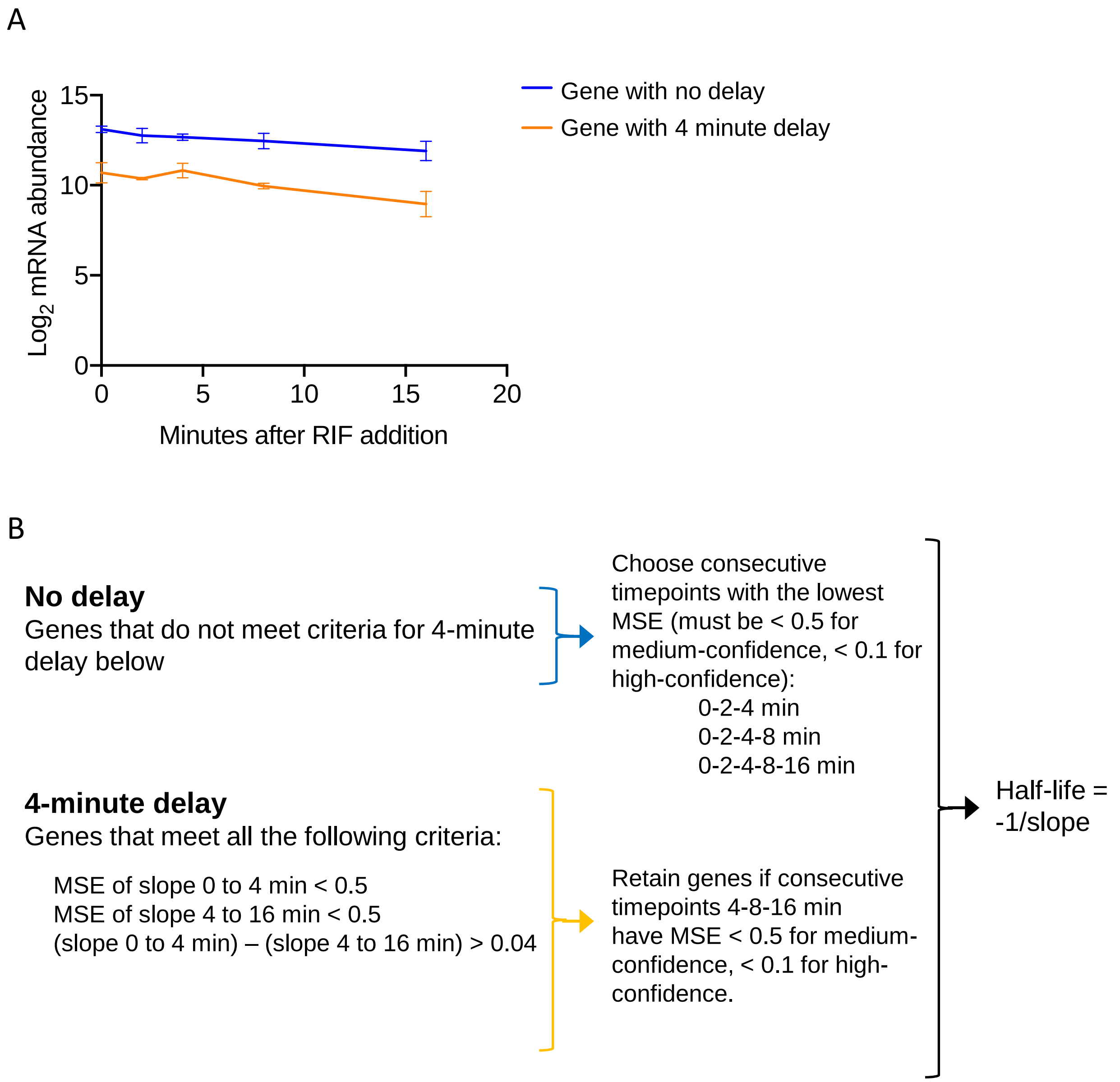

Figure S3. Half-life calculation procedure for genes in *rne* repression condition. A. Log_2-transformed mRNA abundance data show two distinct degradation patterns following addition of rifampicin to block transcription, which can be best categorized as no delay or a 4-minute delay. **B.** Classification of genes into two delay categories based on linear regression fits to different sets of timepoints following addition of rifampicin, followed by half-life determination. The slopes between the indicated timepoints were calculated and used to classify genes as having no delay, or a 4-minute delay. After removal of early timepoints as indicated to account for the delay, the mean squared error (MSE) was used to quantify goodness of fit for linear regression using subsets of the remaining timepoints and the set of timepoints with the best fit were used to calculate the half-life.

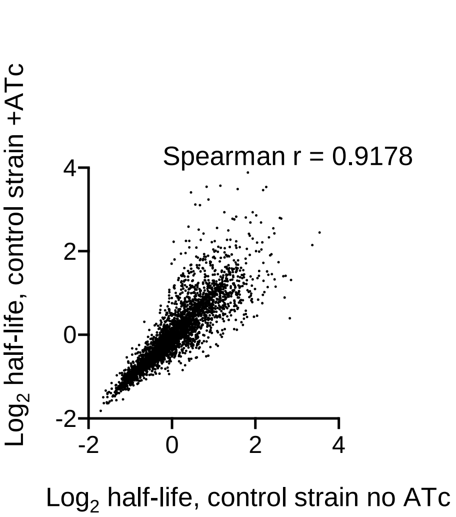

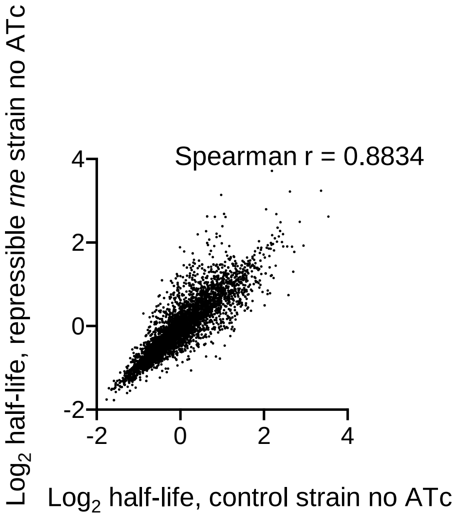

Figure S4. Correlations of half-lives between control conditions. Scatterplots show the half-lives calculated for genes in the control strain with and without ATc (left) and for the repressible and control strains without ATc (right).

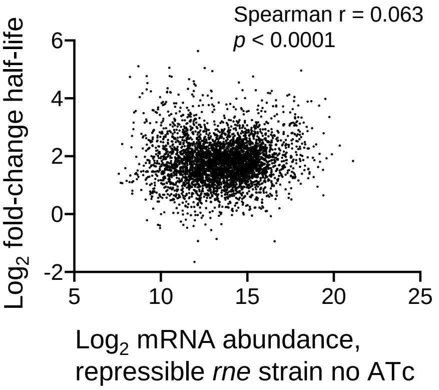

Figure S5. Fold-increase in half-life upon *rne* repression has a very weak correlation with abundance prior to repression. Each dot represents a gene for which half-lives were determined in the presence and absence of ATc in the repressible *rne* strain.

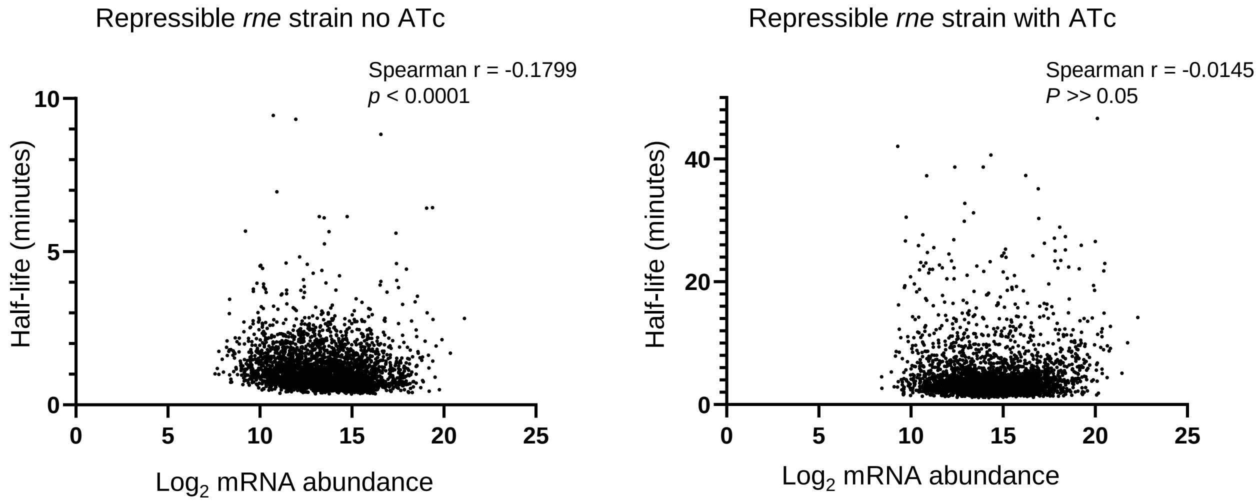

Figure S6. The relationship between mRNA abundance and mRNA half-life changes upon *rne* knockdown. Each dot represents a gene for which half-lives were determined in both the presence and absence of ATc in the repressible *rne* strain.

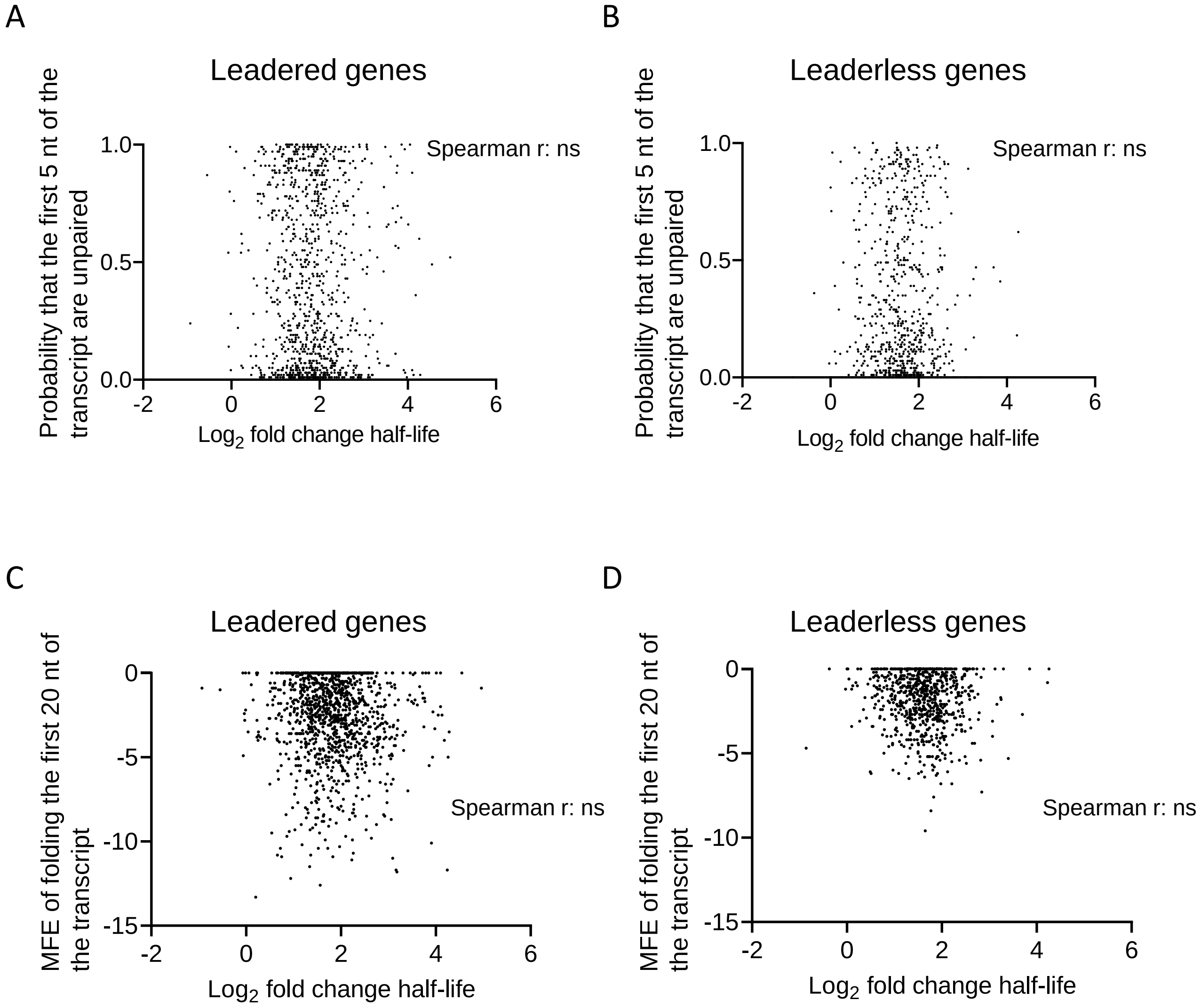

Figure S7. Predicted secondary structure near transcript 5' ends is not correlated with degree of stabilization upon *rne* repression. Each dot represents a gene for which half-lives were determined in the presence and absence of ATc in the repressible *rne* strain. The MFE structure was predicted for the first 20 nt of each transcript (the 5' 20 nt of the 5' UTR for leadered transcripts, and the first 20 nt of the coding sequence for leaderless transcripts). **A** and **B**, the probabilities of the first 5 nt of the transcript being unpaired given the given predicted MFE structures were determined. **C** and **D**, the MFE of folding was determined. All analyses were done in the Vienna RNAfold package (Lorenz *et al*., 2011). ns, $P > 0.05$.

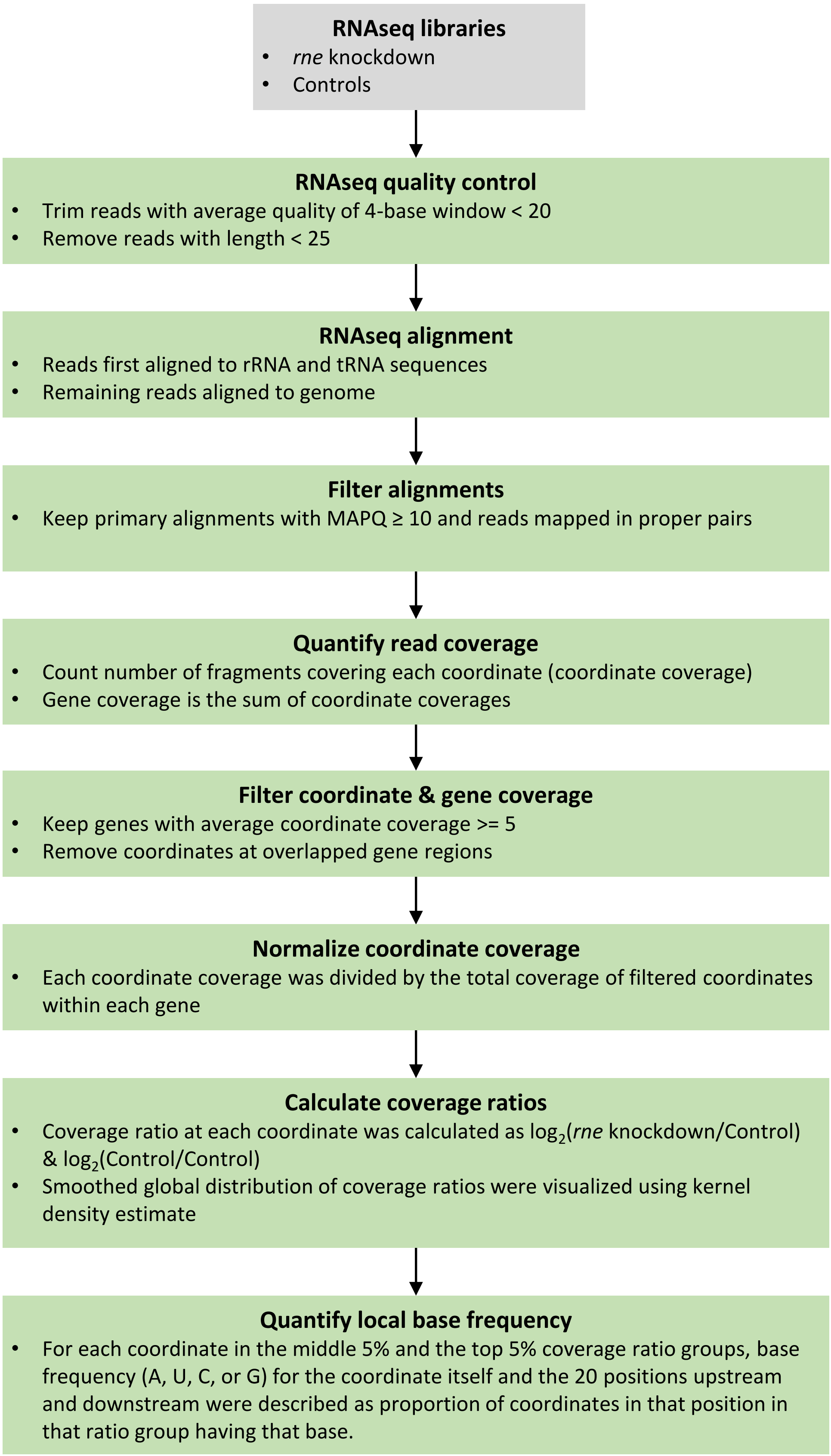

Figure S8. Pipeline for identifying RNase E cleavage sites from standard Illumina RNAseq expression libraries.

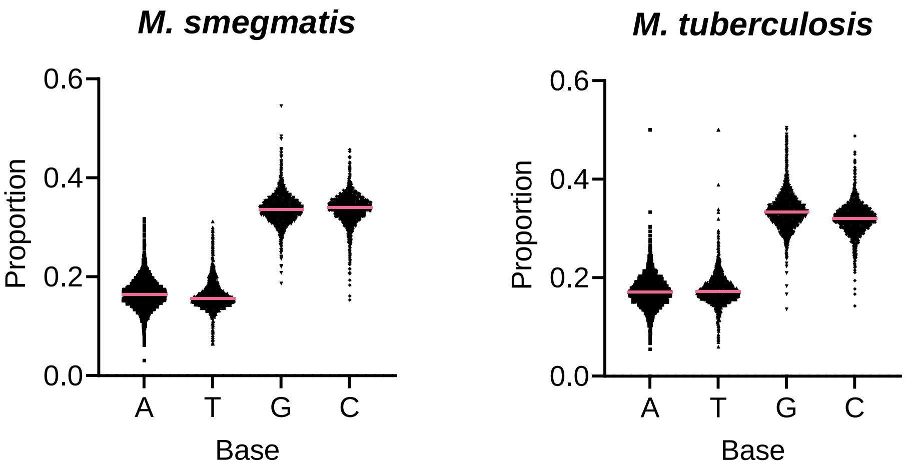

Figure S9. Base composition of coding sequences in *M. smegmatis* and *M. tuberculosis*. Each dot represents a gene. Pink lines indicate medians.

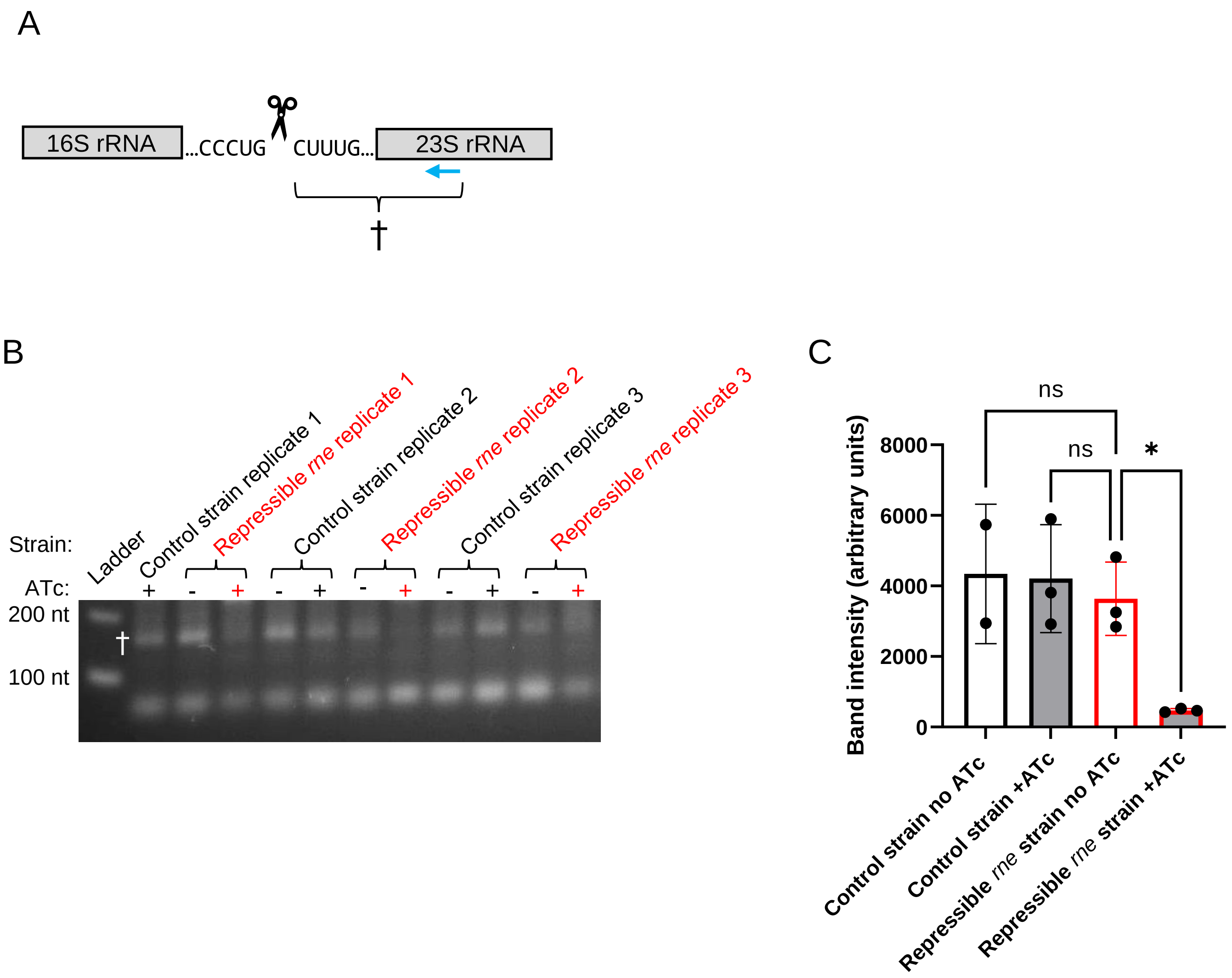

Figure S10. RNase E cleaves upstream of a cytidine during rRNA processing. A. Schematic of part of the rRNA operon with the sequence of a region reported to be cleaved by RNase E shown (Taverniti et al 2011). Graphic not to scale. The scissors indicate the exact cleavage position that we mapped by 5' RACE, which is between positions 2041 and 2042 relative to the start of the rRNA operon (numbering as in Taverniti et al 2011). The cleavage site lies in a region predicted to be single-stranded, approximately 81 nt downstream of a predicted RNase III cleavage site and approximately 27 nt upstream of another predicted RNase III cleavage site (Taverniti et al 2011). The dagger (†) indicates the 5' RACE PCR product shown in panel B. The blue arrow indicates the primer used for cDNA synthesis. **B.** An ethidium-bromide strained agarose gel revealing 5' RACE PCR products. The dagger (†) indicates the PCR product shown schematically in panel A. Triplicate samples are indicated. Control strain replicate 1 in the absence of ATc was not run on this gel. The results are representative of two independent experiments. **C.** Image J was used to quantify the integrated pixel intensity of the band indicated with the dagger (†) in panel B. Strains and conditions were compared by ANOVA and Dunnett's multiple comparisons test. * indicates $p < 0.05$.

5’-AGGGCGCUGAUCGCCAUGUUCCCCUGGUACAUCCAGUGGUUCCCCAACGC^CGUGUGGAAG-3’

5’-ACCUUCGA^CCUGUUCGUCGGCCUCAUCCAGGCCUUCAU^CUUCUCGCUGCUGACGAU^CC-3’

5’-UGUACUUCAGCCAGUCGAUGGAACUGGACCACGAGGACCACUGACGAGCAACCCUGCUGGA-3’
3’-AUGAAGUCGGUCAGCUACCUUGACCUGGUGCUCCUGGUGACUGCUCGUUGGGACGACCU-5’

5’-CCGAACAAAUCCCUACGACCCGAUCGACACGAACUCUGACGGCAACA-3’
3’-GGCUUGUUUAGGGAUGCUGGGCUAGCUGUGCUUGAGACUGCCGUUGU-5’

Figure S11. In vitro-transcribed partial duplex RNA substrate used for RNase E cleavage assays. Black font indicates the sense strand corresponding to the 3’ 159 nt of the *M. smegmatis atpB* coding sequence and 64 nt of the intergenic region between *atpB* and *atpE*. Blue font indicates an antisense strand used to block RNase E cleavage. Cleavage sites mapped in Fig. 5 are shown by red carets.

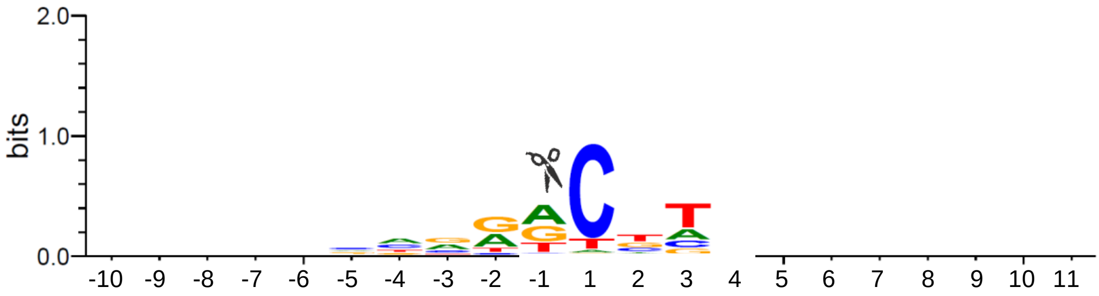

Figure S12. Sequence context of an expanded set of *M. tuberculosis* RNA cleavage sites. 10,795 putative cleavage sites were identified using relaxed filters as described in the methods section. A weblogo (Weblogo 3.7.12) was constructed with a background frequency of 65% G+C.

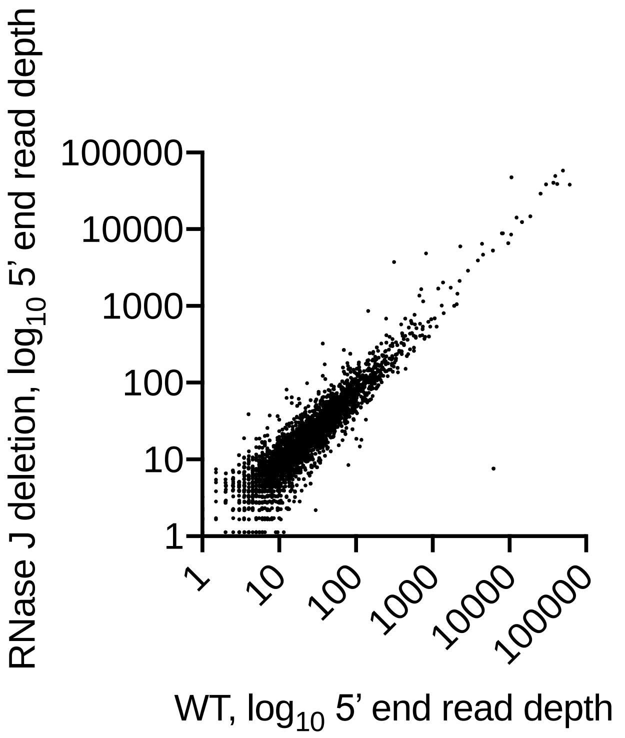

Figure S13. Most *M. tuberculosis* cleavage sites have similar abundance in WT H37Rv and an isogenic strain in which the gene encoding RNase J was deleted. Monophosphorylated RNA 5' ends were mapped and quantified by adapter ligation and Illumina sequencing. Read depth for each 5' end produced by the cleavage sites listed in Supplementary Table 5 is shown.

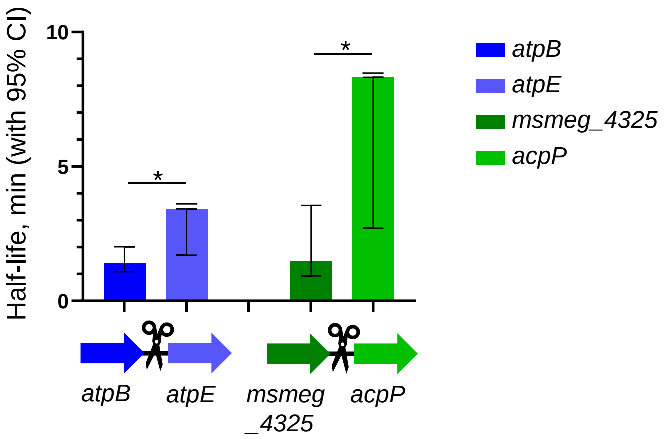

Figure S14. *M. smegmatis* gene pairs that appear to be co-transcribed and are bisected by cleavage sites display differential stabilities. Abundance of the four indicated transcripts was measured by qPCR 0, 4, and 8 minutes after addition of rifampicin to block transcription, and half-lives were calculated by linear regression of log_2-transformed abundance. Error bars show the 95% confidence intervals of each half-life. The top error bar was truncated in two cases (*atpE* and *acpP*) where the upper 95% CI was infinity. *, $p < 0.05$ for comparison of the half-lives of the indicated genes by linear regression.
